# Characterization and root cause analysis of immunogenicity to pasotuxizumab (AMG 212), a prostate-specific membrane antigen-targeting bispecific T-cell engager therapy

**DOI:** 10.3389/fimmu.2023.1261070

**Published:** 2023-10-23

**Authors:** Hweixian Leong Penny, Kelly Hainline, Nathaniel Theoharis, Bin Wu, Christian Brandl, Christian Webhofer, Mason McComb, Sabine Wittemer-Rump, Gökben Koca, Sabine Stienen, Ralf C. Bargou, Horst-Dieter Hummel, Wolfgang Loidl, Carsten Grüllich, Tobias Eggert, Ben Tran, Daniel T. Mytych

**Affiliations:** ^1^ Department of Clinical Immunology, Amgen, Thousand Oaks, CA, United States; ^2^ Labcorp, Translational Biomarker Solutions, Greenfield, IN, United States; ^3^ Department of Biologics, Amgen, Thousand Oaks, CA, United States; ^4^ Department of Translational Safety & Bioanalytical Sciences, Amgen Research (Munich) GmbH, Munich, Germany; ^5^ Department of Process Development, Amgen Research (Munich) GmbH, Munich, Germany; ^6^ Department of Clinical Pharmacology, Modeling & Simulation, Amgen, Thousand Oaks, CA, United States; ^7^ Bayer AG, Research and Development Oncology (RED Onc), Pharmaceuticals, Berlin, Germany; ^8^ Department of Early Development (Oncology), Amgen Research (Munich) GmbH, Munich, Germany; ^9^ Translational Oncology/Early Clinical Trial Unit (ECTU), Comprehensive Cancer Center Mainfranken, University Hospital Wurzburg, Wurzburg, Germany; ^10^ Department of Urology, Ordensklinikum Linz GmbH, Linz, Austria; ^11^ Department of Medical Oncology, National Center for Tumor Diseases, Heidelberg University Medical Center, Heidelberg, Germany; ^12^ Department of Early Development (Oncology), Amgen, Thousand Oaks, CA, United States; ^13^ Department of Medical Oncology, Peter MacCallum Cancer Centre, Melbourne, VIC, Australia

**Keywords:** immunogenicity, BiTE^®^, T cell engager, ADA, prostate cancer

## Abstract

**Introduction:**

In oncology, anti-drug antibody (ADA) development that significantly curtails response durability has not historically risen to a level of concern. The relevance and attention ascribed to ADAs in oncology clinical studies have therefore been limited, and the extant literature on this subject scarce. In recent years, T cell engagers have gained preeminence within the prolific field of cancer immunotherapy. These drugs whose mode of action is expected to potently stimulate anti-tumor immunity, may potentially induce ADAs as an unintended corollary due to an overall augmentation of the immune response. ADA formation is therefore emerging as an important determinant in the successful clinical development of such biologics.

**Methods:**

Here we describe the immunogenicity and its impact observed to pasotuxizumab (AMG 212), a prostate-specific membrane antigen (PSMA)-targeting bispecific T cell engager (BiTE®) molecule in NCT01723475, a first-in-human (FIH), multicenter, dose-escalation study in patients with metastatic castration-resistant prostate cancer (mCRPC). To explain the disparity in ADA incidence observed between the SC and CIV arms of the study, we interrogated other patient and product-specific factors that may have explained the difference beyond the route of administration.

**Results:**

Treatment-emergent ADAs (TE-ADA) developed in all subjects treated with at least 1 cycle of AMG 212 in the subcutaneous (SC) arm. These ADAs were neutralizing and resulted in profound exposure loss that was associated with contemporaneous reversal of initial Prostate Surface Antigen (PSA) responses, curtailing durability of PSA response in patients. Pivoting from SC to a continuous intravenous (CIV) administration route remarkably yielded no subjects developing ADA to AMG 212. Through a series of stepwise functional assays, our investigation revealed that alongside a more historically immunogenic route of administration, non-tolerant T cell epitopes within the AMG 212 amino acid sequence were likely driving the high-titer, sustained ADA response observed in the SC arm.

**Discussion:**

These mechanistic insights into the AMG 212 ADA response underscore the importance of performing preclinical immunogenicity risk evaluation as well as advocate for continuous iteration to better our biologics.

## Introduction

Biologics such as monoclonal antibodies and their associated bispecific antibody constructs consist of large and complex structures. Some of these amino acid sequences and structural motifs, may induce humoral immune responses due to non-self recognition by the patient’s immune repertoire, resulting in the formation of specific anti-drug antibodies (ADAs).

The ADA response is initiated by antigen-presenting cells (APCs) that phagocytose, internalize, and process the drug into smaller peptides. These peptides are loaded onto major histocompatibility complex (MHC) class II at the APC cell surface for presentation to CD4+ T cell clones that recognize the specific peptide-MHCII complex (pMHC) (1–3). At the same time, B cells recognizing structural motifs in the tertiary structure of the protein therapeutic are stimulated to produce IgM. However, IgM responses are often transient. For a sustained humoral response, B cells must be further activated to differentiate into plasma cells, which subsequently affinity mature and isotype class-switch to become potent IgG producers. This additional “help” is accomplished mainly by CD4+ T cells which have been activated by pMHC recognized on the APC (1–3). Therefore, sustained ADA formation is a coordinated response engaging several immune cell types: APC capture, processing and presentation; B cell recognition of conformational epitopes and T cell recognition of sequence-based epitopes from the same antigen.

ADAs can cause unintended clinical consequences affecting exposure and safety, with effects ranging from none to life-threatening. ADAs may impact the pharmacokinetics (PK) of a drug (4–6), maintaining or more often, decreasing, exposure depending on whether the ADAs are sustaining or clearing antibodies respectively (7). Even though ADAs can affect PK, this does not necessarily translate to impaired efficacy of the drug. Patients risk experiencing reduced efficacy particularly in cases where early-onset, high magnitude, high-affinity neutralizing ADAs (NAb) are induced (4–6). NAbs bind to the variable regions of the antibody to prevent engagement of the target antigen, effectively stymying therapeutic activity. In contrast, binding ADAs that bind to other parts of the antibody, such as the Fc region, may not directly result in loss of therapeutic activity. However, both binding and neutralizing ADAs may lead to formation of large drug-antibody immune complexes that can be rapidly cleared by phagocytes in the spleen and liver, resulting in suboptimal exposure and eventual loss of efficacy (4–7). In oncology, ADAs may rise to the level of concern when there is potential for clear differences in key Response Evaluation Criteria in Solid Tumors (RECIST) response parameters such as progression-free survival (PFS) and overall survival (OS) between ADA-positive and ADA-negative subgroups. Patients in whom ADAs develop are also at increased risk for certain adverse events such as complement-mediated reactions, infusion-related reactions, or other Type III hypersensitivity events due to the deposition of these immune complexes in microvessels (8).

In addition to treatment-induced ADAs, pre-existing reactivity has been detected in drug-naïve individuals (9). While the origin of pre-existing reactivity is not well-understood, and the clinical impact of which can be highly variable, pre-existing reactivity represents an additional layer of immunogenicity monitoring when evaluating novel protein therapeutics. However, clinically significant pre-existing reactivity against biologics is rare and is not often boosted upon dosing with investigational drug.

In oncology, the risk of ADA development may be lower in patients due to their disease state itself, or in patients who have recently completed chemotherapy and whose immune systems may still be recovering from these myeloablative regimens. The suppressed ability to mount a robust antibody response may have accounted for the historically low rates of ADAs observed to tumor-associated antigen targeted, monoclonal antibody-based investigational drugs.

Clinical immunogenicity has gained renewed interest of late, as immunotherapies exhibiting ability to potently trigger an immune response have brought the topic of drug-induced immunogenicity into active discourse. In a focused review consolidating immunogenicity data from 81 clinical trials with anti-cancer biologics, Van Brummelen and colleagues observed that 63% of these studies report ADA formation (10), suggesting that many compounds currently being investigated in oncology are potentially immunogenic. However, the clinical relevance of some of these ADAs remain unclear.

In a similar assessment, Davda and colleagues reviewed the incidence of ADA and NAb across multiple, approved, anti-cancer antibody-based immunomodulatory agents and found that the data is suggestive of a higher likelihood of immunogenicity to antibodies with T cell or APC targets compared to B cell targets (11). Not surprisingly, in a more recent review focused on bispecific antibody constructs in the immuno-oncology (IO) space, Zhou and colleagues reappraise the need for immunogenicity risk assessment throughout development for this class of biologics and have provided specific recommendations (12). Taken together, this underscores the need for close monitoring of potential immunogenicity to drugs being advanced in IO studies, particularly drugs which target T cell priming and activation.

Pasotuxizumab (henceforth referred to as AMG 212) is a 55 kD protein with an anti-PSMA target binding domain linked to an anti-CD3 binding domain. It is a Bispecific T cell engager (BiTE^®^) molecule, a class of biologics whose mode of action is such that when the BiTE^®^ molecule is bound on one end to target protein on the surface of a target cell and bound to CD3 on a T cell at the other end, proximity-induced, redirected T cell lysis of target cells can occur. In NCT01723475, a FIH study, AMG 212 was tested in mCRPC patients, who were refractory to novel anti-androgen therapy (abiraterone and/or enzalutamide) and had failed at least one (but not more than two) taxane regimen.

It has been previously reported that anti-androgen therapy can modulate the immune milieu in the tumor microenvironment, promoting an immunosuppressed state in mCRPC patients. However, Gardner and colleagues showed that a vaccine based on a novel recombinant soluble PSMA protein was able to elicit anti-PSMA antibodies in patients with progressive prostate cancer (13). These antibodies reacted strongly with prostate cancer cells and increased with multiple dosing. Taken together, the data show that despite immunomodulation by prior therapy, along with tumor escape and immune resistance mechanisms in this patient population, mCRPC patients are still capable of mounting an antigen-specific humoral response to a biologic.

Here we report the clinical immunogenicity to AMG 212 and its impact as observed in NCT01723475. We characterize how ADA onset, magnitude and kinetics impacted the PK, pharmacodynamic (PD) response and adverse events observed on study. We also describe a measure implemented mid-study to mitigate the ADAs detected in the SC arm. Further, we performed a root cause analysis to explain the immunogenicity observed, by assessing potential contributing factors such as the baseline immune status of subjects and product quality attributes of the drug lots administered to subjects. Finally, through a series of *in vitro* assays, we identified non-tolerant sequence-based epitopes contributing to the robust and clinically impactful ADA response to AMG 212 delivered subcutaneously.

## Materials and methods

### AMG 212 study design

This was an open-label, multicenter, Phase I, dose-escalation study (ClinicalTrials.gov, NCT01723475) conducted at five clinical study centers in Germany and Austria and sponsored by Bayer AG, Leverkusen, Germany. It was designed to determine the safety and maximum tolerated dose (MTD) of AMG 212 (primary objectives) and to assess pharmacokinetics, PSA and tumor response (secondary objectives) and biomarkers (exploratory objective) of AMG 212 administered either by daily SC injection or CIV infusion. An independent data monitoring committee was established to regularly review safety data. The starting doses for the SC and CIV arms were 0.5 and 5 μg daily, respectively. A cycle for this study, in both the SC and CIV arms, was defined as 21 days (or 3 weeks).

In the SC arm, AMG 212 was administered daily by SC injection, with no breaks between cycles. The SC dosing schema is shown in **Supplementary Figure S1A**. SC injection sites included four abdominal regions around the navel, upper arm and thighs. The 2 ml syringes for SC administration were prepared by the local pharmacy and administered either in the clinic by a healthcare professional or at home by the patient.

In the CIV arm, AMG 212 was administered as a continuous IV infusion, using an on-body portable infusion pump and central venous port system. In the first 4 cycles (first 12 weeks on study), patients received treatment on a “5 week on-1 week off” schedule, whereby AMG 212 was administered over 5 weeks, followed by a treatment-free interval of 1 week. From cycle 5 onwards, patients could continue treatment on the “5 week on-1 week off” schedule or switch to a “4 week on-2 week off” schedule, at the discretion of the investigator and the subject. The CIV dosing schema is shown in **Supplementary Figure S1B**.

In both the CIV arm and the higher dose-level SC cohorts, prophylactic oral or IV dexamethasone was administered before the administration of AMG 212 to mitigate cytokine release syndrome (CRS) risk. At the discretion of the investigators, concomitant therapy was allowed. For each patient, treatment continued until tumor progression, unacceptable toxicity, consent withdrawal, or withdrawal from the study. Further details on study design can be found in Hummel et al., 2021 (14).

### AMG 212 patients

Men aged ≥ 18 years old with histologically or cytologically confirmed advanced CRPC with treatment failure after ≥ 1 taxane regimen and who were refractory to abiraterone and/or enzalutamide or refused any other standard therapy were eligible for inclusion in the study. Eligible patients had undergone bilateral orchiectomy or received continuous androgen deprivation therapy and had evidence of progressive disease after discontinuation of anti-androgen therapy (i.e., flutamide, bicalutamide or nilutamide) before study drug treatment. Additional inclusion and exclusion criteria have been described in Hummel et al., 2021 (14).

### AMG 160 study design

This was an open-label, multi-center, phase 1, dose-exploration/dose-expansion study in patients with mCRPC from North America, Europe, Asia, and Australia (NCT03792841) and sponsored by Amgen. The primary objectives of the study were to evaluate the safety and tolerability of AMG 160, to determine the MTD and/or recommended phase 2 dose (RP2D). The secondary objectives were to evaluate the preliminary antitumor activity and characterize the PK and pharmacodynamics of AMG 160.

AMG 160 was administered intravenously in 28-day cycles. Once the target dose was reached, AMG 160 was administered by short-term IV infusion over 1 hr, every 2 weeks. In the dose exploration phase, to mitigate against CRS, step-dosing was implemented. Similar to the premedications used in the AMG 212 study, prophylactic oral and/or IV dexamethasone was administered before the administration of AMG 160 to mitigate CRS risk.

Following the dose exploration phase, a dose expansion study was conducted to confirm the safety, PK, and pharmacodynamics of AMG 160 at the RP2D, and to obtain further safety and efficacy data and carry out correlative biomarker analysis.

### AMG 160 patients

The AMG 160 patient population enrolled in NCT03792841 was comparable to that enrolled for AMG 212 in NCT01723475. In brief, men aged ≥18 years of age were included if they had histologically or cytologically confirmed mCRPC that was refractory to novel hormonal therapy, had failed 1–2 taxane regimens (or were unsuitable for or had refused treatment with taxanes), and had evidence of progressive disease as defined by the Prostate Cancer Working Group 3 (PCWG3) guidelines. Patients were excluded if they had active autoimmune disease or required immunosuppressive therapy, had received prior PSMA–targeted therapy (patients treated with PSMA radionuclide therapy were considered eligible), or had evidence of central nervous system metastases, leptomeningeal disease, or spinal cord compression.

### AMG 212 and AMG 160 patients

Both the AMG 212 and AMG 160 clinical studies were conducted in accordance with the ethical principles derived from international guidelines, including the Declaration of Helsinki, Council for International Organizations of Medical Sciences International Ethical Guidelines, and applicable International Conference on Harmonisation guidelines, laws, and regulations. The study protocols were approved by the Institutional Review Board/Independent Ethics Committee at each study site. All patients provided written informed consent.

### Anti-AMG 212 antibody assessments (binding and neutralizing antibody assays)

#### ADA sampling timepoints

In the SC arm, blood samples for immunogenicity evaluation were collected predose on Cycle 1 Day 1, 8 and 15, on Day 1 of each cycle from Cycle 2 to 8, on Day 1 of every second cycle thereafter and at least 36 hr after the last dose of AMG 212. In the CIV arm, blood samples were collected predose on Cycle 1 Day 1, 8 and 15, on Day 1 of each subsequent cycle and at least 36 hr after the last dose of AMG 212. The ADA collection schedule for the SC and CIV arms is shown in **Supplementary Figures S1A, B**.

#### Binding ADA assay

Anti-AMG 212 antibodies were measured using a validated electrochemiluminescence-based bridging assay. This immunoassay method followed a two-tiered assay approach consisting of a screening assay and confirmatory assay. Samples were diluted 1:10 in D-PBS (Gibco Cat# 14190-094) or D-PBS and soluble drug (confirmatory assay only) prior to analysis. The samples were then incubated with conjugate mixture consisting of biotinylated-AMG 212 and ruthenylated-AMG 212. During this incubation, the two antigen binding sites of anti-AMG 212 antibodies were able to form a bridge between the labeled AMG 212 molecules. The sample mixture was then added to a blocked streptavidin microtiter plate, washed, and analyzed on a plate reader. The result was a series of electrically induced oxidation-reduction reactions involving ruthenium (from the captured complex) and tripropylamine. In this immunogenicity screening assay, a subject-specific floating cut point was calculated by adding a specific normalization factor to the pre-dose subject sample. Samples with results equal to or greater than the assay cut point were then tested to confirm specificity of the response. Samples classified as positive in the confirmatory assay were further titrated in 10% human serum pool and reported at the highest dilution titer at which a positive response was determined. The normalization factor and the confirmatory assay cut point were calculated from 40 prostate cancer donor serum samples. The assay sensitivity was 8.6 ng/mL based on a goat polyclonal positive control antibody. At 12, 120 and 1,200 ng/mL of anti-AMG 212 antibody, the assay tolerated at least 10,000 pg/mL of excess AMG 212.

#### Neutralizing ADA assay

The resulting immunoassay-positive samples were analyzed using a cell-based bioassay to determine whether the detected binding antibodies have neutralizing properties. This AMG 212 neutralizing assay was based on an *in-vitro* cell-based competitive ligand binding assay. Effector cells (CD3-positive MC15 cells) were incubated with target cells (human PSMA-positive C4-2 cells), serum samples and AMG 212. After overnight incubation, the cytotoxic activity was measured with the luminescent CytoTox-Glo™ Cytotoxicity Assay kit (Promega). The CytoTox-Glo™ Cytotoxicity Assay uses a luminogenic peptide substrate, the AAF-Glo™ Substrate, to measure dead-cell protease activity released from cells that have lost membrane integrity. When the serum sample contained AMG 212 neutralizing antibodies, cytotoxicity was reduced. A sample was considered positive for neutralizing antibodies if the decrease of the cytotoxicity was greater than the cut point compared to the maximal toxicity sample. The cut point was calculated from 45 healthy donor serum samples. The assay sensitivity was 780 ng/mL based on a goat polyclonal positive control antibody. At 13,500 ng/mL of anti-AMG 212 antibody, the assay tolerated at least 3,000 pg/mL of excess AMG 212.

### AMG 212 pharmacokinetics assessment

#### PK sampling timepoints

In the SC arm, blood samples for PK assessment were collected predose at Cycle 1 Day 1 and 15, and at 1, 2, 4, 6, 8, 12 and 24 hr post-infusion at these two timepoints. Samples were also collected predose on Cycle 1 Day 3, 4 and 8. From Cycle 2 to 8, and every second cycle thereafter, a blood sample was collected on Day 1 within 2 to 6 hr post-dose. In the CIV arm, blood samples were collected on Cycle 1 Day 1 predose, at the 2-3 hr and 4-6 hr post-start of infusion timepoints, and on Day 2, 8 and 15. From Cycle 2 onwards, a blood sample was collected on Day 1 and 15 with every subsequent even cycle, and collected on Day 1, 2 and 15 with every subsequent odd cycle.

#### PK assay

The assay to quantify AMG 212 was based on a sandwich immunoassay format in which capture antibodies (goat polyclonal anti-AMG 212 antibodies) were coated on a plate. After sample incubation, a mouse anti-idiotype monoclonal antibody against the CD3 binding domain of AMG 212 was bound to the captured AMG 212 and detected with ruthenylated anti-mouse antibody. The assay range was 0.150 to 111 ng/mL.

### AMG 212 pharmacodynamic assessments (PSA and peripheral blood immune cell biomarkers)

Efficacy was assessed according to the Prostate Cancer Clinical Trials Working Group 2 recommendations (15). RECIST responses are not described in this manuscript but were reported in Hummel et al., 2021 (14). Changes in serum PSA levels were assessed predose on days 1, 8 and 15 of cycle 1, at the beginning of each subsequent cycle and at the end of treatment visit. A PSA response was defined as a 50% reduction in the PSA level from baseline that was confirmed by a second test value at least 3 weeks later. Other pharmacodynamic markers, including peripheral blood biomarkers of T-cell activation (including CD69) and monocyte activation (including HLA-DR) were assessed by flow cytometry and were conducted before and during treatment.

### Epibase® MHC class II-associated peptide proteomics assay

The Epibase® MAPPS assay was performed at Lonza. Monocytes were isolated from frozen PBMC samples by positive magnetic bead selection (Miltenyi Biotec). Monocytes were seeded into T12.5 flasks with 5 X 10^6^ monocytes per flask in differentiation medium (Dendritic Cell (DC) medium containing 100 ng/mL IL-4, 50ng/mL GM-CSF) and incubated for 5 days at 37°C, 5% CO2 to differentiate into DC. The DC were then loaded with AMG 212 (or medium alone for the Blank) and matured with Lipoprotein polysaccharide (LPS) for 24 hr. After maturation, the DC were lysed and the membrane fraction containing the HLA:peptide complexes was solubilized and incubated with Protein A mag sepharose beads (GE Healthcare) coated with anti-HLA-DR antibody (Lonza) at 4°C overnight. The following morning the beads were washed in Tris Buffer Solution (TBS) and the peptides eluted from the HLA-DR complex with 0.1% Trifluoroacetic acid (TFA). Finally, the peptides were purified by passing through a 10kDa Molecular Weight Cut-Off (MWCO) spin column and stored at -80°C for mass spectrometry (MS) analysis. MS data analysis was carried out using the PEAKS® Studio Software package. The identified peptides are then mapped back to the full-length AMG 212 protein sequence and compared to an Epibase® in silico analysis to identify which HLA-DR alleles are responsible for the peptide binding. Albumin was also included as an internal control in the samples to verify assay performance. Comparable numbers of total (173 vs 171) and distinct (85 vs 77) albumin peptides were detected in the AMG 212 sample in the first and second round of MAPPS respectively, indicating that the assay was consistent and sensitive across repeats.

### Test and control peptides

Along with the MAPPS-identified sequences (#1-5, #8, #8.5, 11), peptides spanning the rest of the Complementarity Determining Regions (CDR) regions of AMG 212 were proactively synthesized. These additional sequences were labeled Peptide #6, 7, 9, 10 and 12. A separate peptide, Peptide #13, was synthesized as a known self-tolerant peptide and acted as a negative control peptide that demonstrated MHC class II-binding but was not expected to confer T cell reactivity. A total of 14 peptides were synthesized.

Pool 1 consisted of 5 peptides (Peptide #1, 2, 3, 4 and 5) and Pool 2 consisted of 8 peptides (#6, 7, 8, 9, 10, 11, 12 and 13). Peptide #8.5 was inadvertently missed in Pool 2. CEFTA, a peptide pool consisting of 35 MHC class II-restricted peptides from human CMV, EBV, influenza virus, tetanus toxin, and adenovirus 5, and PADRE (Pan DR-binding epitope), were used as positive controls as these peptides are designed to stimulate T cells with a broad array of HLA types.

### Restimulated T cell line assay

Isolated CD4+ T cells were stimulated with multiple rounds of autologous monocyte-derived DCs (moDCs) pulsed with our test peptides in a 4-week co-culture. For the first 3 weeks, the T cells were stimulated weekly with freshly-derived moDCs pulsed with peptide pools in the first 3 stimulations, and then with individual peptides from a pool, for a fourth and final stimulation.

On Day 1, CD14+ cells were isolated from PBMC using positive selection magnetic microbeads (Miltenyi Biotech). The CD14+ cells were differentiated into immature DCs by seeding at 1 X 10^6^/mL into a 96 well plate (200 µL/well) in Cellgenix DC GMP Medium (Sartorius) supplemented with 100 ng/mL IL-4 (Peprotech) and 50 ng/mL GM-CSF (Peprotech). After 5 days, the immature DCs were separately loaded with 5 µg/mL CEFTA peptide (Mabtech) pool, 5 µM PADRE peptide (Mayflower Biosciences), or 5 µM test peptide pool and matured with 10 ng/mL TNF-α (R&D Systems) and 5 ng/mL IL-1β (Peprotech) for 48 hours. The quality of the matured DCs was assessed by labeling of markers HLA-DR, CD14, CD80, CD83, CD86, CD209, and CD11b. CD4+ T cells were isolated from PBMC using negative selection magnetic microbeads (Miltenyi Biotech). CD4+ T cells (2 X 10^5^/well) were stimulated by peptide-loaded DC and cultured initially in AIM-V supplemented with 2% Human AB Serum (Sigma-Aldrich) for 21 days. Freshly loaded and matured DCs were added to the T cell culture every 7 days, and the culture medium was refreshed every 7 days with AIM-V supplemented with 2% Human AB Serum (Sigma-Aldrich), 10 U/mL IL-2 (Peprotech), and 5 ng/mL IL-7 (Peprotech).

On Day 21, a fraction (4-5 X 10^4^) of CD4+ T cells were taken from each well and stimulated with peptide pool-loaded DCs in pre-coated Human Interferon-γ PVDF Plates (ImmunoSpot®). After 48 h incubation, the manufacturer’s plate development instructions were followed to detect secreted IFN-γ. Spots were counted using a CTL ImmunoSpo® S6 Ultra M2 Analyzer. Wells with unloaded DCs (absence of peptide, but with T cells) and wells with T cells only (no DCs, no peptide), served as negative controls. T cell lines reactive to individual peptides were determined as wells that had spot counts at least two-fold higher in the presence of peptide compared to the unloaded DC negative control, with a minimal difference of 30 spots, as described previously (16, 17). The identified antigen-specific T cell lines were then divided and stimulated with individual peptide-loaded DCs in the fourth week of co-culture. The same ELISpot protocol was applied, and the same parameters were used to determine if a T cell line was specific to an individual peptide (2-fold higher than negative controls, with a minimal difference of 30 spots).

### Clinical memory recall assay

Ten ml of whole blood at the end-of-treatment (EOT) timepoint was collected per subject, according to the Schedule of Assessments in the AMG 160 FIH Study 20180101 (NCT03792841). The blood was sent ambient on the same day of collection to the central lab for PBMC processing using the CTL protocol (18) within a 48 hr window from time of collection. The PBMCs were enumerated and stored in liquid nitrogen at a cell concentration of 10 X 10^6^/ml before onward batch shipment to Labcorp Translational Biomarker Solutions. Upon thawing of the patient PBMCs, we noted poor viability and functionality in the majority of samples. Thus, we performed a pilot experiment to bulk stimulate a known reactive donor’s PBMCs using a test peptide pool of our suspect sequences to clonally expand peptide-specific T cell memory clones over 10 days. This was a strategy undertaken previously by other groups in non-oncology disease indications (19). However, our efforts were unsuccessful in sustaining viability of these mCRPC patient PBMCs despite providing multiple cytokines to stimulate growth and proliferation such as IL-2, IL-4, and an anti-CD28 antibody.

Ultimately, to perform the clinical memory recall assay, freshly-thawed patient PBMCs were seeded at 2 X 10^5^/well into pre-coated Human Interferon-γ PVDF Plates (ImmunoSpot®) and incubated with individual test peptides at 5 µM each, in CTL-Test Medium supplemented with 2mM GlutaMAX and 10 ng/mL of GM-CSF (Peprotech). The peptides tested were #1, 2, 8, 8.5, 11, along with peptides #13 and #4 which we had established previously from the restimulated T cell line assays to be negative controls (did not demonstrate T cell reactivity). Phytohemagglutinin (PHA) at 2 µg/mL was used as a strong, non-specific stimulator for a technical positive control. PBMCs from an AMG 160-naive healthy donor, acted as an additional negative control for the assay. After 72 hr of incubation (37°C, 5% CO2, humidified chamber), manufacturer’s plate development instructions were followed to detect secreted IFN-γ. Spots were counted using a CTL Immunospot® Series 5 Macro Analyzer.

### Statistical analysis

Analyses of AMG 212 immunogenicity, PK and signs of activity were descriptive in nature and presented using summary statistics and individual subject profiles. ADA status, exposure impact and PSA response correlation analyses were performed using a logistic regression model with SAS (version 9.4) software. Further details on sample sizes, dose-limiting toxicities (DLTs) and safety summary statistics to fulfill the study objectives can be found in a prior clinical report summarizing the overall results of the AMG 212 FIH study (14). Patients who completed the study without any major protocol violations were included in the PK, ADA, PD and where applicable, PSA response evaluation sets. In figures where specific parameters are being compared between the SC and CIV subjects, statistical significance was determined by the Student’s t-test (two-tailed), whereby p values of < 0.05 were considered significant and denoted by a single asterisk*, and n.s. refers to “not significant”.

## Results

### Incidence, magnitude, and kinetics of TE-ADA to AMG 212 in the SC arm

NCT01723475 was first initiated in a cohort of 31 subjects who were subcutaneously administered AMG 212 into four regions around the navel (other injection sites were permitted) (14). Doses were given daily for 21 days per cycle, at dose levels ranging from 0.5 µg to 172 µg per day until confirmed disease progression if there were no other reasons to discontinue AMG 212 treatment. At appropriate timepoints, patient serum samples were collected and screened for binding and neutralizing antibodies to AMG 212.

Pre-existing reactivity was not observed to AMG 212. However, as an aggregate across all doses in the SC dose escalation study, treatment-emergent ADA (TE-ADA) developed in 30/31 subjects (96.7%) who had a post-baseline result (**Table 1.1**). None of the ADAs developed on-study were transient (**Table 1.1**). Except for one subject who did not receive AMG 212 past Cycle 1 Day 8, all 30 subjects who completed at least one treatment cycle of AMG 212 developed TE binding ADAs (30/30, 100% incidence). Of these 30 binding ADA-positive subjects, all except two with very low titer ADA (1:30 and 1:90), had binding ADA that was also neutralizing (28/30, 93.3% incidence). Therefore, the validated neutralizing antibody assay used to test AMG 212 clinical study samples had sufficient drug tolerance and was sensitive enough to capture almost all subjects positive for binding ADA.

**Table 1.1:** Anti-AMG 212 Antibody Incidence in Subcutaneous (SC) Arm.

	Cohort 1 0.5 µg/d (N = 1)	Cohort 2 1.5 µg/d (N = 1)	Cohort 3 4.5 µg/d (N = 1)	Cohort 4 9.0 µg/d (N = 3)	Cohort 5 18 µg/d (N = 3)	Cohort 6 36 µg/d (N = 4)	Cohort 7 72 µg/d (N = 3)	Cohort 8 144 µg/d (N = 3)	Cohort 9 172 µg/d (N = 3)	Cohort 10 172 µg/d + GC (N = 6)	Cohort 11 144 µg/d + GC (N = 3)	Total (All cohorts) (N = 31)
Subjects with a result at baseline	1	1	1	3	3	4	3	3	3	6	3	31
Pre-existing Ab incidence - n (%)												
Binding antibody positive at baseline	0/1 (0.0)	0/1 (0.0)	0/1 (0.0)	0/3 (0.0)	0/3 (0.0)	0/4 (0.0)	0/3 (0.0)	0/3 (0.0)	0/3 (0.0)	0/6 (0.0)	0/3 (0.0)	0/31 (0.0)
Subjects with a postbaseline result	1	1	1	3	3	4	3	3	3	6	3	31
Treatment-emergent Ab incidence - n (%)												
** *Binding*** antibody positive postbaseline with a negative result at baseline	1/1 (100.0)	1/1 (100.0)	1/1 (100.0)	3/3 (100.0)	3/3 (100.0)	4/4 (100.0)	3/3 (100.0)	2/3 (66.7)	3/3 (100.0)	6/6 (100.0)	3/3 (100.0)	30/31 (96.7)
** *Neutralizing*** antibody positive postbaseline with a negative result at baseline	1/1 (100.0)	1/1 (100.0)	1/1 (100.0)	3/3 (100.0)	3/3 (100.0)	4/4 (100.0)	2/3 (66.7)	2/2 (100.0)	2/3 (66.7)	6/6 (100.0)	3/3 (100.0)	28/30 (93.3)
Transient ^a^	0/1 (0.0)	0/1 (0.0)	0/1 (0.0)	0/3 (0.0)	0/3 (0.0)	0/4 (0.0)	0/3 (0.0)	0/2 (0.0)	0/3 (0.0)	0/6 (0.0)	0/3 (0.0)	0/30 (0.0)

N = Number of subjects who received ≥ 1 dose of investigational productAb = Antibody. n = number of subjects with a result. ^a^ Negative result at the subject's last timepoint tested within the study period. GC = topical glucocorticoid treatment at SC injection sites. **Tables 1.1** and **1.2** has been previously reported as Supplementary Tables S5 and S6 respectively in Hummel et al., 2021 (14).

**Figure 1:**
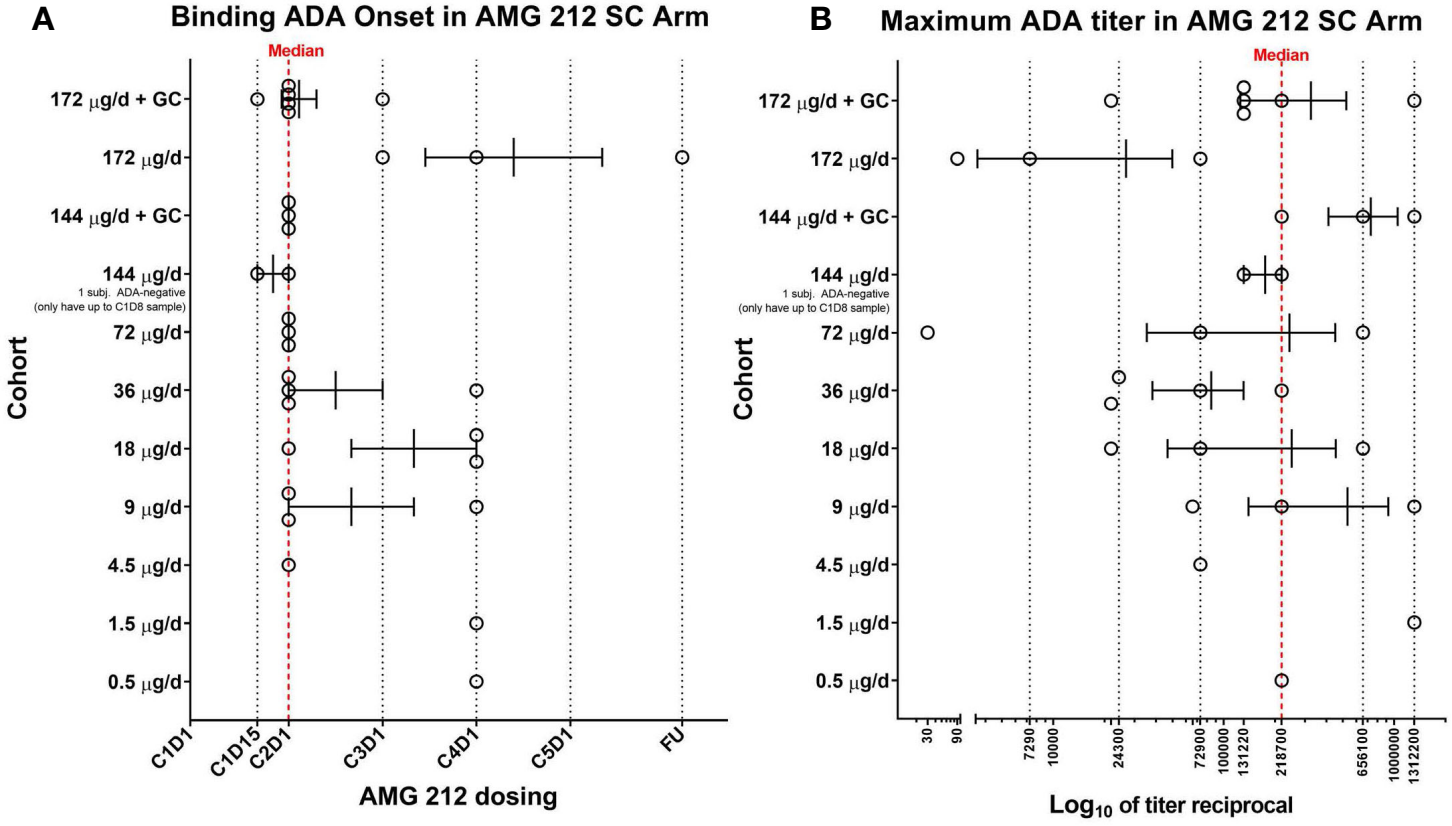
Binding ADA onset and maximum ADA titer in the SC arm of the AMG 212 first-in-human study. At appropriate timepoints, patient serum samples were collected and screened for binding antibodies to AMG 212 using a fully validated, electrochemiluminescence-based antibody assay. 30 of the 31 subjects enrolled in the SC arm completed at least 1 cycle of AMG 212 SC dosing. All 30 subjects developed binding ADA and are shown in the scatter plots **(A, B)**. Each circle represents a single subject. Each row represents individual cohorts, starting from Cohort 1 (bottom, 0.5 µg/d) to Cohort 10 (top, 172 µg/d + topical glucocorticoid (GC) treatment at the SC injection sites). Subjects were enrolled in single-subject cohorts for the first 3 cohorts and in multiple-subject cohorts thereafter. The scatter plot in **(A)** shows the range of binding ADA onset in each cohort, plotted as cycle number, day number (CXDX) upon initiation of AMG 212 dosing. FU refers to the 30-day follow-up period after the end of treatment. Error bars depict the mean and standard error of mean (SEM) of the binding ADA onset within each cohort. The red dotted line at Cycle 2 Day 1 (or Day 22) represents the median binding ADA onset across the dose escalation phase in the AMG 212 SC arm. The scatter plot in **(B)** shows the range of maximum ADA titer in each cohort, plotted as the reciprocal of the maximum ADA titer registered by each subject at any time on study. Error bars depict the mean and SEM of the maximum ADA titer reciprocal within each cohort. The red dotted line at titer reciprocal 218700 represents the median maximum ADA titer across the dose escalation phase in the AMG 212 SC arm.

The majority of ADAs had an onset spanning Cycle 1 Day 15 to Cycle 4 Day 1, with median onset at Cycle 2 Day 1, or Day 22 (**Figure 1A**) (14). Ten subjects had ADAs whose maximum titers were achieved at follow-up (FU) (data not shown). Since patients in the SC arm were treated for an overall median time of 91 days, this indicated that peak ADA titers were not yet reached even at the final scheduled antibody collection timepoint tested, about 3 months from the initiation of AMG 212 dosing. The median ADA titer across the 30 binding ADA+ subjects was 218, 700 (**Figure 1B**). Nineteen of the 31 subjects had approximated ADA concentrations above 1 µg/ml (Signal-to-Noise, S/N extrapolated using goat anti-AMG 212 antibody positive control). Both measures of ADA magnitude (titer and S/N) indicate that the ADA responses in these patients were significant. The ADA response was not dose-dependent i.ea higher ADA incidence, an earlier onset of ADA development, or a greater magnitude of ADA, was not observed at higher dose levels compared to lower dose levels (**Figures 1A, B**).

Taken together, TE-ADA observed in the subcutaneous cohort (i) developed early, within the first 2 cycles of AMG 212 treatment, (ii) often progressed to high titers that neutralized AMG 212 activity and (iii) was sustained till end of study as none of the ADAs were transient.

### TE-ADA in the SC arm: clinical impact to exposure, PSA response and safety

Exposure-ADA correlation analyses were performed to determine the impact of the TE-ADA on exposure and efficacy. Following subcutaneous administration, PK was not consistently detectable at lower dose levels. Therefore, it was not reasonable to make any PK-ADA associations at these lower dose levels.

From the 72 µg to 172 µg dose level, PK was detectable in all subjects at first. However, of the 17 TE-ADA+ subjects, 14 subjects had PK samples measuring below the lower limit of quantitation (LLOQ) and 3 subjects had PK samples measuring close to LLOQ, either at the same timepoint as the first positive ADA sample, or at timepoints thereafter (**Supplementary Figures S2A, B**). This profound loss of exposure, which coincided with ADA onset, was most likely due to the development of TE-ADA that cleared AMG 212 to undetectable levels in these subjects.

**Figure 2:**
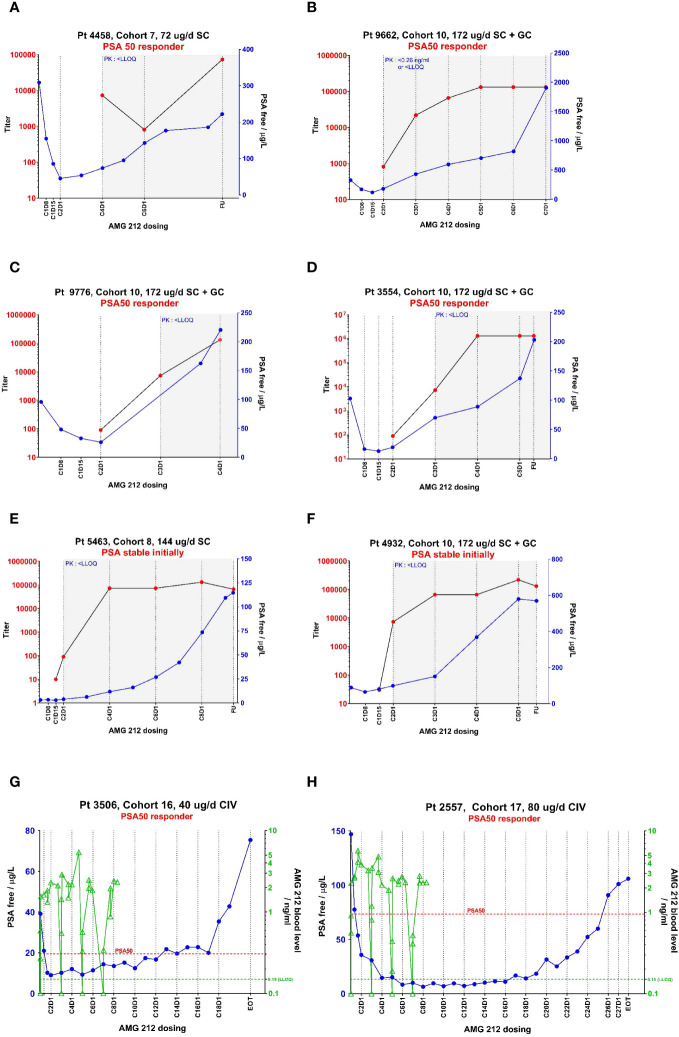
PK, ADA and PSA profiles from select individual subjects in the AMG 212 SC and CIV arm showing temporal correlation between these 3 parameters. The temporal relationship between pharmacokinetics (PK, as represented by drug concentration), ADA magnitude (as represented by titer) and a pharmacodynamic marker of biochemical disease progression (as represented by Prostate Surface Antigen (PSA)), are plotted in line graphs **(A–H)**. CXDX refers to the cycle number and day number upon initiation of AMG 212 dosing. “EOT” refers to End-of-Treatment. FU refers to the 30-day follow-up period after the end of treatment. **(A–F)** show the profiles of six subjects from the SC arm, which include four PSA 50 responders **(A–D)** and two PSA stable subjects **(E, F)**. **(G, H)** show the profiles of two subjects from the CIV arm who were both PSA 50 responders. In the SC arm, AMG 212 PK was not consistently detectable among subjects in the same cohort until Cohort 7, the 72 µg/d dose level. At Cohort 7 and onwards, while PK was detectable initially, the onset of ADAs correlated with an impact to PK, whereby PK fell to below the lower limit of quantitation (LLOQ). The time period at which PK measured <LLOQ is depicted by a gray shaded area on the graphs **(A–F)**. In the CIV arm, ADAs did not develop in all treated subjects, and PK was detectable in all cohorts (no gray shaded areas in **(G, H)**). In **(A–F)**, ADA titer is shown on the left y-axis, and PSA on the right y-axis. In G-H, PSA is shown on the left y-axis and PK on the right y-axis. In **(G, H)** the PK trace stops at Cycle 8 Day 8 for both Subject 3506 and Subject 2557, as that was the last PK timepoint collected for these patients on study. The legend for the line graphs is as follows - PK: green triangles 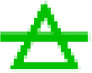, ADA-positive status: red circles 
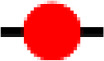
, PSA: blue circles 
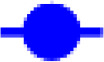
; green dotted line at 0.15 ng/ml is the LLOQ of the PK assay; red dotted line represents the PSA value at which 50% reduction from baseline was observed.

A key pharmacodynamic (PD) marker in prostate cancer is Prostate Surface Antigen (PSA), which acts as a clinically validated marker of disease progression and therefore a surrogate marker for drug activity. From the 36 to 172 µg dose level, reductions in PSA > 50% relative to baseline were observed in nine patients (14). In these initial PSA responders, ADA-mediated loss of exposure likely resulted in an elimination of initial PSA decline, with subsequent progressing PSA. Four examples of such subjects are shown in **Figures 2A–D**. These examples show contemporaneous association of ADA onset with drug clearance, an ensuing rise of PSA and loss of drug activity gains made in the first cycle. In addition, 3 subjects who had stable PSA initially, also recorded rising PSA levels upon developing ADA. Two such examples are shown in **Figures 2E, F**. In total, of the 14 ADA+ subjects who had an exposure impact (PK<LLOQ at or after ADA onset), 13 had a PSA rebound from an initial PSA decline or PSA stable status (**Supplementary Figure S2C**).

Given the high titers of ADAs observed in the SC arm, the ADAs were assessed for any association with immune-complex related safety events known to be associated with ADAs, such as infusion reactions or other hypersensitivity events (8). While infusion reactions and hypersensitivity events were not reported, out of 30 ADA-positive subjects, 24 were observed to develop injection site reactions (14). These were localized injection site erythemas indicating cutaneous inflammation. However, there were 6 ADA-positive subjects that did not develop injection site reactions. Therefore, based on the analysis of this small sample size, an association of these ADAs with injection site reactions could not be identified.

Taken together, TE-ADA observed in the SC cohort was not clearly associated with adverse events but did result in uniform exposure loss. This most likely accounted for the curtailment of the PSA response observed initially.

### Topical glucocorticoid co-treatment at SC injection sites to mitigate ADAs

**Figure 3:**
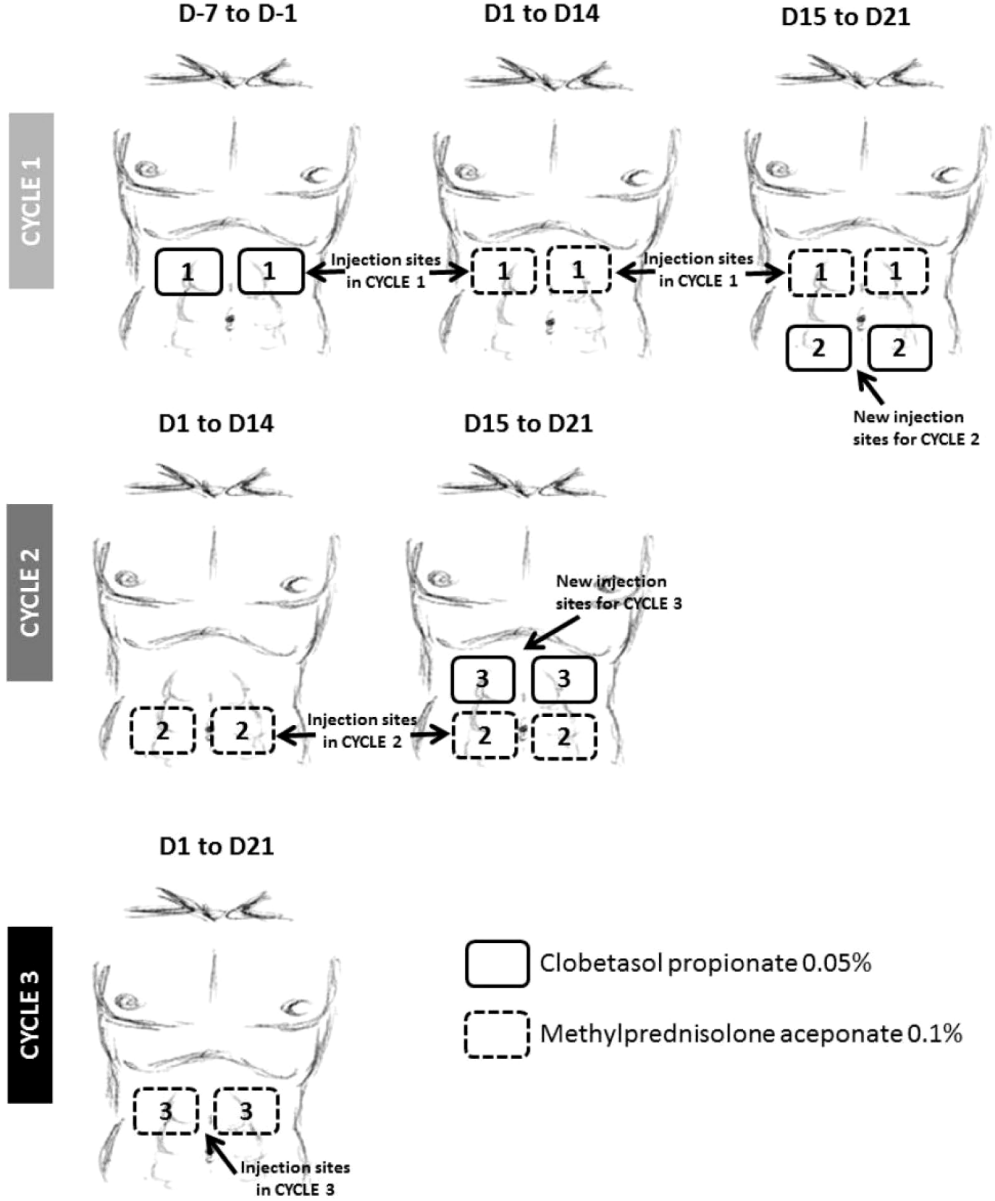
Topical glucocorticoid (GC) treatment at AMG 212 SC injection sites implemented at the 144 and 172 µg/d dose levels. To mitigate the ADA observed during dose escalation, a daily topical GC treatment of SC injection sites for the first 3 cycles was introduced mid-study, with the goal of suppressing skin antigen presenting cell (APC) function. The above schema provided instructions on administering the topical GC in patients who injected AMG 212 at 4 regions around the navel. On Cycle 1, Day minus 7 to Cycle 1 Day minus 1, i.e. 1 week to 1 day prior to start of AMG 212, subjects applied a hazelnut-sized amount of clobetasol propionate 0.05% cream in a uniform layer on each of 2 abdominal skin areas for a 7-day daily topical administration. Upon initiation of the AMG 212 SC daily dosing cycle, from Cycle 1 Day 1 to Cycle 1 Day 21, subjects continued applying daily topical GC on the same 2 marked skin areas where SC injections were performed, with methylprednisolone aceponate 0.1% cream. The AMG 212 SC injection was always performed before the administration of the topical GC on the same day. This “7-day clobetasol premedication, 21-day methylprednisolone concomitant medication” topical GC regimen was repeated for Cycle 2 and Cycle 3, on 2 other abdominal skin areas distinct from the injection sites of the previous cycle. A subject stopped administration of topical GC if a local reaction related to the AMG 212 SC injections or a Grade ≥2 local or systemic reaction related to GC treatment occurred. Further daily injections were then performed outside the marked skin areas selected for the ongoing cycle.

In the SC cohort, due to the consistent formation of ADAs and the observation of localized injection site reactions in many ADA-positive subjects, it was hypothesized that ADA development was induced by the skin-resident DCs, which are well-established to be excellent APCs (20, 21). To prevent, reduce and/or delay ADA development, topical glucocorticoid (GC) treatment including clobetasol propionate and methylprednisolone was introduced mid-study in a protocol amendment, due to their known ability to reduce DC numbers and inhibit their function (22–24). Parallel cohorts of subjects dosed at the 144 µg (Cohort 11) and 172 µg (Cohort 10) dose levels received an aggressive regimen of topical GC (**Figure 3**). Designated injection sites at the abdomen were pre-treated with clobetasol propionate 0.05% cream for 7 days before the Cycle 1 Day 1 AMG 212 SC dose to induce apoptosis of skin APCs. The same injection sites were then further treated with methylprednisolone aceponate 0.1% cream during the 21-day cycle to suppress APC function. This was repeated through the third cycle (**Figure 3**).

The ability of the topical GC to eliminate or suppress skin APCs was not confirmed, as skin biopsies were not retrieved from GC-treated subjects. As a surrogate marker of whether the topical GC eliminated or suppressed skin APCs, peripheral blood monocyte counts were analyzed. There were no significant differences in this parameter, at the 144 and 172 µg/d dose levels, between the paired cohorts comparing subjects treated with or without GC (**Supplementary Figures 3A, B**).

Despite utilizing topical GC to forestall APC engagement, this strategy was not successful in preventing, delaying or suppressing the magnitude of ADA development (**Table 1.1**, comparing Cohort 10 to Cohort 9, and Cohort 11 to Cohort 8; **Figures 1A, B**: top 4 rows).

### TE-ADA in the CIV arm

As the ADAs observed in the SC cohort could not be mitigated, it was not feasible to continue dose escalation in the SC setting as exposure could not be reasonably sustained past the second cycle. A new arm of the study testing AMG 212 via continuous intravenous (CIV) administration was initiated. In stark contrast to the SC cohort, 0/16 subjects (0% incidence) developed ADAs in the CIV cohort when administered AMG 212 at dose levels ranging from 5 µg/d to 80 µg/d (**Table 1.2**). This result was not due to false negatives, as the drug tolerance for the ADA assay was satisfactory and drug interference could be ruled out. As expected, in these ADA-negative subjects, clinically-observed exposure was sustained, dose-proportional, and fell within the normal range of variability (Supplementary Figure S1 of Hummel et al., 2021 (14)).

**Table 1.2:** Anti-AMG 212 Antibody Incidence in Continuous Intravenous Infusion (CIV) Arm.

	Cohort 13 5 µg/d (N = 3)	Cohort 14 10 µg/d (N = 4)	Cohort 15 20 µg/d (N = 3)	Cohort 16 40 µg/d (N = 4)	Cohort 17 80 µg/d (N = 2)	Total (All cohorts) (N = 16)
Subjects with a result at baseline	3	4	3	4	2	16
Pre-existing Ab incidence - n (%)						
Binding antibody positive at baseline	0/3 (0.0)	0/4 (0.0)	0/3 (0.0)	0/4 (0.0)	0/2 (0.0)	0/16 (0.0)
Subjects with a postbaseline result						
Treatment-emergent Ab incidence –n (%)						
** *Binding*** antibody positive postbaseline with a negative or no result at baseline	0/3 (0.0)	0/4 (0.0)	0/3 (0.0)	0/4 (0.0)	0/2 (0.0)	0/16 (0.0)
Transient ^a^	0/0 (–)	0/0 (-)	0/0 (-)	0/0 (-)	0/0 (-)	**0/16** (0.0)

N = Number of subjects who received ≥ 1 dose of investigational product.

Ab = Antibody.

n = number of subjects with a result.

a Negative result at the subject’s last timepoint tested within the study period.

GC = topical glucocorticoid treatment at SC injection sites.

**Tables 1.1** and **1.2** has been previously reported as Supplementary Tables S5 and S6 respectively in Hummel et al., 2021 (14).

From the 5 to 80 µg/d dose levels, confirmed PSA responses (PSA 30 or PSA 50) were recorded in 5/16 subjects (14). Out of these 5 PSA responders, 3 subjects had an initial PSA decline that was reversed, despite sustained exposure in the absence of TE-ADA. These 3 subjects were dosed at lower dose levels at which a sustained pharmacodynamic response from AMG 212 may not be expected, and for which other resistance mechanisms may have played a role in the observed loss of response as well. Remarkably, 1 subject each at the two highest dose levels tested, 40 µg/d and 80 µg/d, had sustained PSA 50 responses for 12 cycles and 25 cycles respectively (**Figures 2G, H**).

Taken together, in contrast to the SC route, AMG 212 did not induce any TE-ADAs when administered by CIV infusion. This enabled maintenance of exposure, yielding an exceptional durability of response in two subjects at the higher dose levels.

### Root cause analysis of the difference in clinical immunogenicity observed between the SC and CIV cohorts

Several factors contribute to a therapeutic’s immunogenic risk and observed immunogenicity in the clinic. We sought to explain the polar difference in TE-ADA incidence observed between the SC (near 100%) and CIV (0%) cohorts by systematically interrogating both product-related and patient-specific factors, beyond the route of administration.

### Immune status of SC and CIV subjects

**Figure 4:**
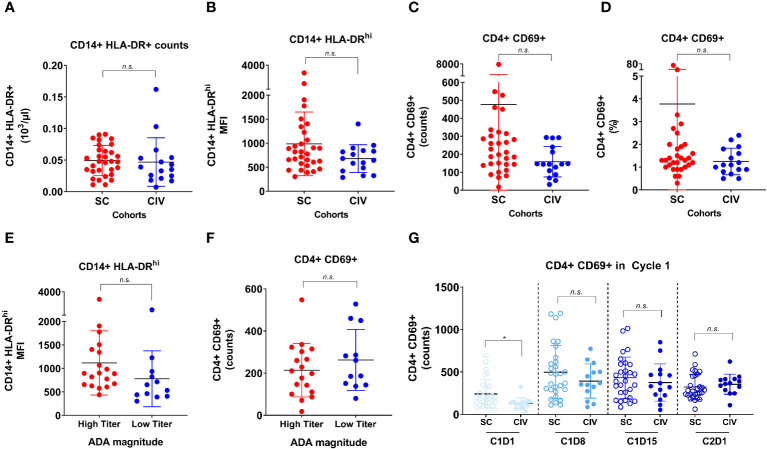
Flow cytometric analysis of CD14+ monocyte and CD4+ T cell activation between SC and CIV arms of AMG 212. MHC class II upregulation on CD14+ monocytes, as evaluated by HLA-DR+ counts and HLA-DR^hi^ median fluorescence intensity (MFI) **(A, B)**, and the activation status of CD4+ T cells, as evaluated by CD69+ counts and CD69+ cells as a percentage of CD4+ T cells **(C, D)**, were assessed by flow cytometry in peripheral blood at the time of screening (7 days before cycle 1 day 1). ADA+ subjects in the SC arm were further sub-grouped into those who had a maximum ADA titer corresponding to more than (high titer) or less than (low titer) of 1: 10, 000 at any time on study **(E, F)**. Peripheral CD4+ T cell activation status between the SC and CIV arms was analyzed at predose timepoints through the first cycle (on day 1, 8, 15) and on cycle 2 day 1 **(G)**. Each circle represents an individual subject. Unpaired t tests were used to compare between the SC and CIV subjects. n.s. is not significant. *p-value < 0.05.

To determine if an elevated baseline immune status in the SC subjects played a role in predisposing them to developing ADA, activation status (CD69+) on CD4+ T cells and MHC class II upregulation (HLA-DR^hi^) on monocytes were assessed by flow cytometry in peripheral blood at the time of screening (7 days before Cycle 1 day 1). At the screening timepoint, no significant differences in CD14+ HLA-DR+ counts (**Figure 4A**), HLA-DR^hi^ median fluorescence intensity (MFI) (**Figure 4B**), CD4+ CD69+ counts (**Figure 4C**) and percentages (**Figure 4D**), were observed between SC and CIV subjects. TE-ADA+ subjects in the SC cohort were further spliced into those who had a maximum ADA titer corresponding to more than (high titer) or less than (low titer) of 1: 10, 000, at any time on study. Subjects with high titer TE-ADA did not exhibit significantly greater monocyte MHC class II upregulation (**Figure 4E**) or T cell activation (**Figure 4F**) compared to those with low titer TE-ADA at screening. Taken together, the data suggest that SC subjects were not inadvertently biased to developing TE-ADA from higher predose immune parameters relevant to generating an ADA response that may have occurred by chance.

In addition, as most of the TE-ADA developed by the end of cycle 1, we assessed peripheral CD4+ T cell activation at all timepoints in the first cycle for which flow cytometry data was available. Although CIV subjects had lower CD4+CD69+ counts at baseline compared to SC subjects, the CD4+ T cell activation status did not appear to be increased in SC subjects compared to CIV subjects over the period of Cycle 1 Day 8 to Cycle 2 Day 1, when the majority of TE-ADA developed (**Figure 4G**). Flow cytometry assessing B cell markers of activation was not performed in this study. Thus, CD4+ T cell activation documented over time in the peripheral blood, was unable to capture the ongoing ADA response generated in the secondary lymphoid tissue.

### Product quality attributes of Good Manufacturing Practice lots used in the SC and CIV arms

Of a drug product’s various attributes, high molecular weight species (HMWS) (larger than dimer) is an attribute widely acknowledged as a primary risk to immunogenicity (25–27). The AMG 212 SC and CIV Good Manufacturing Practice (GMP) lots were formulated the same as a lyophilisate, for reconstitution with sterile water for injection (WFI). Upon review of the drug product quality profile of AMG 212 GMP lots, drug product monomer purity was comparable at 97-98% in each of the SC and CIV GMP lots, indicating that HMWS levels were low and comparable between the lots used in the SC and CIV arms of the study (**Table 2**). Other product quality attributes with immunogenicity risk potential, such as visible particles, and particulate matter, including the pH of the formulations, were comparable between the SC and CIV GMP lots as well (**Table 2**). Taken together, the drug product quality attribute data suggest that SC subjects were not inadvertently biased to developing TE-ADA due to higher amounts of immunogenicity-risk related attributes in the SC GMP lots that may have occurred by chance.

**Table 2:** Comparison of selected drug product quality attributes related to immunogenicity risk between SC and CIV lots used in the AMG 212 study.

Attribute	SC GMP Lot #1	SC GMP Lot #2	CIV GMP Lot #1	CIV GMP Lot #2
Appearance, visible particles	Free from particles	Free from particles	Free from particles	Free from particles
Subvisible particles (per container)				
*≥ 25 µm*	0	1	1	0
*≥ 10 µm*	3	4	9	15
Purity, % monomer by SE-HPLC	98	97	98	97
pH-value	6.0	5.9	6.0	6.2

Attributes known to potentially contribute to immunogenicity risk were evaluated to rule out differences between the SC and CIV GMP lots that could have accounted for the disparity in ADA incidence between the two arms. The attributes of (1) visible particles, (2) subvisible particles, (3) AMG 212 drug product purity (% monomer by size exclusion-high performance liquid chromatography (SE-HPLC) and (4) pH-value are shown in **Table 2**. A near 100% drug product purity (% monomer by SE-HPLC) indicate low levels of other size variants including high molecular weight species (HMWS). Both the SC and CIV GMP lots passed acceptance criteria for these attributes and are considered comparable to each other.

In addition, we examined stability assays that tested whether AMG 212 was stable over time after reconstitution. AMG 212 consisted of over 97% monomer species as measured by size-exclusion chromatography, after 7 days at 5 ± 3°C, and an additional 4 days at 37 ± 2°C with agitation (data not shown). This data suggests that it was unlikely that AMG 212 drug product could have developed HMWS in concerning amounts over time at human body temperature for at least 4 days. Taken together, an assessment of HMWS in AMG 212 drug substance and drug product ruled out this attribute as a potential cause for the immunogenicity observed in the SC arm.

Given the above, patient baseline immune status (peripheral blood), and drug product quality attributes related to immunogenicity risk, did not appear to contribute to ADA formation in SC-administered subjects.

The immunogenicity observed to SC-dosed AMG 212 may be most evidently explained by the route of administration. However, relying on this factor alone would be an oversimplification, as not all SC-injected protein therapeutics above 20kD in size, which are known to first encounter the lymphatic system before entering the peripheral circulation (28, 29), elicit ADA responses. **Table 1.1** shows that TE-ADAs developed in every dose level of the SC cohort, from the lowest to the highest dose level tested. This indicates a lack of dose-dependency in the induction of the ADA response. It also suggests that a characteristic inherent in the drug may be driving immunogenicity.

To determine where the AMG 212 SC ADAs were binding to on AMG 212, we performed exploratory work evaluating the domain specificity of these ADAs. Using 8 ADA-positive and 14 ADA-negative samples from 4 subjects (1 subject each from the 0.5, 1.5, 4.5 and 9.0 µg/d cohorts) in an exploratory assay and with appropriate reagents, these results demonstrated that AMG 212 ADAs bound predominantly to the PSMA binder, and not to the CD3 binder or the linker (data not shown).

However, sustained, clinically impactful ADA responses such as those observed in the AMG 212 SC arm are often driven not by structural epitopes recognized by B cells alone, but by CD4+ T cells recognizing sequence-based epitopes located within the drug’s amino acid sequence. We therefore focused our efforts on seeking out potential T cell epitope(s) in AMG 212. Here we hypothesized that the combination of the SC drug delivery regimen and the existence of potentially immunogenic sequences in AMG 212 were responsible for driving the robust clinical ADA response.

To address the latter part of this hypothesis, we performed a series of *in vitro* experiments to determine whether AMG 212 contained potentially non-tolerant, sequence-based, T cell epitopes.

### Identification of potential sequence-based epitopes in AMG 212

#### MAPPS, restimulated T cell line assay and clinical memory recall assay

We applied a tiered approach in seeking out potential T cell epitopes in AMG 212. Starting at the level of APC recognition and presentation, we narrowed down suspect sequences through their reactivity in healthy donor T cells, and eventually tested the peptides in clinical memory recall assays using patient samples.

First, we sought to determine whether there were specific sequences in AMG 212 that were being presented on the APC surface to T cells by employing MHC class II-associated peptide proteomics (MAPPS) (30, 31). While MAPPS does not assess the ability of peptide-MHC complexes to elicit a T cell response directly, it seeks to identify MHC class II-binding peptide sequences that are naturally processed and presented by MHC class II on the surface of APCs. These sequences can then be identified by mass spectrometry, allowing for precise location mapping onto the full-length sequence.

**Figure 5:**
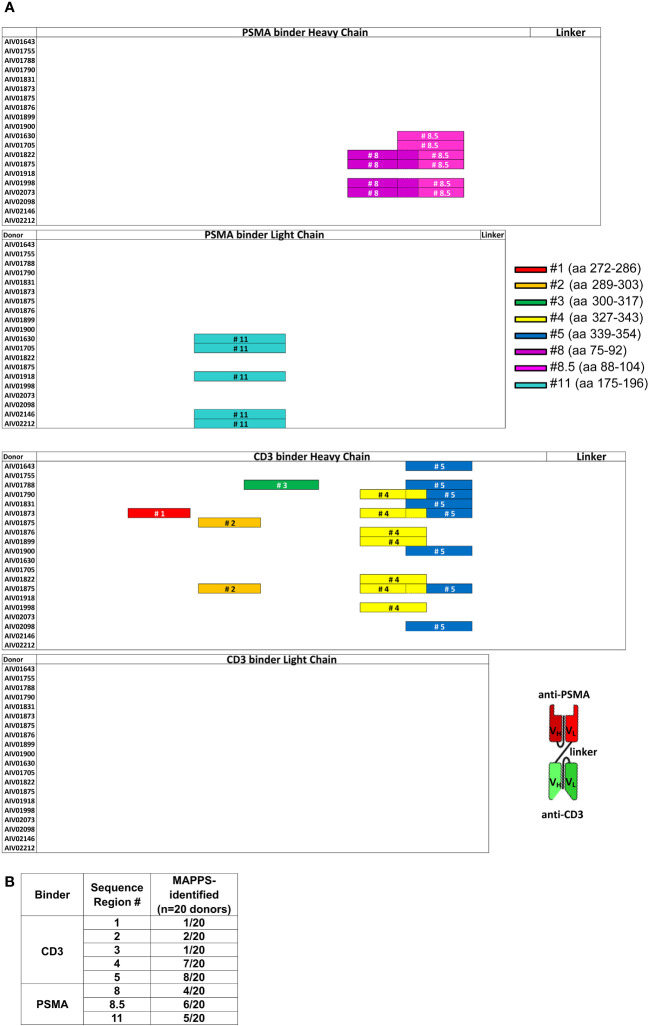
MHC class II-associated peptide proteomics (MAPPS) assay identified sequences within full-length AMG 212 that were naturally processed and presented on the APC surface for presentation to T cells. Immature DCs were loaded with AMG 212 to allow for capture, processing and formation of peptide major histocompatibility complex (pMHC) complexes. Cells were harvested and lysed to immunoprecipitate the pMHC complexes from the DC surface. Peptides were eluted off the presenting MHC class II molecules, and their sequences identified by mass spectrometry, allowing for precise location mapping onto the full-length sequence. **(A)** This sequence map depicts the full-length AMG 212 sequence, divided into four sub-sections: the PSMA binder, heavy and light chains (top half; top two sub-sections) and the CD3 binder, heavy and light chains (bottom half; bottom two sub-sections). Each row within each sub-section represents a single donor. The different color bars mapped onto each of the rows denote the location and length of the distinct sequence regions #1-5, #8, 8.5 and 11. These sequences ranged from 14 – 20 amino acids long and their amino acid (aa) residue numbers (start and end) are shown alongside their respective bars in the legend. The overlap between sequence region #8 and #8.5, #4 and 5, are depicted by a dotted border. Several donors presented multiple peptides within the same sequence region, but a single color bar is shown to account for all sequences within that region that were detected from that donor. A schematic of the overall structure of AMG 212 is shown next to the sequence maps for reference. V_H_ and V_L_ refer to the single chain variable heavy and single chain variable light regions of the antibody construct respectively. MAPPS was performed on AMG 212 twice, evaluating a total of 20 donors that included a variety of HLA-DRB alleles representing the major subtypes in the human population. The table in **(B)** shows the aggregate number of donors that presented each sequence region as an incidence of the 20 donors utilized in the MAPPS assays. The HLA allele subtypes of these 20 donors are found in **Supplementary Table 1**.

MAPPS was performed on AMG 212 twice, evaluating a total of 20 donors that included a variety of HLA-DRB alleles representing the major subtypes in the human population (**Supplementary Table 1**). MAPPS identified 8 distinct sequence regions across the full-length amino acid sequence of AMG 212 that was being presented on HLA-DRB alleles. These were labeled as Sequence Region #1, 2, 3, 4, 5, 8, 8.5 and 11 (**Figure 5A**). Sequence region #1-5 were located in the CD3 binder, while sequence region #8, 8.5 and 11 were located in the PSMA binder of AMG 212 (**Figure 5A**). The overall number of donors that presented each sequence region are shown in **Figure 5B**. Although there were a few sequence regions that appeared to be presented by multiple donors, MAPPS did not reveal any one sequence region as a potentially immunodominant epitope over the rest of the regions based on incidence (**Figure 5B**).

While MAPPS helps to vastly narrow down possibilities of culprit epitopes, this assay does not verify that these putative sequences are immunogenic (32). Therefore, each putative sequence region identified by MAPPS needed to be confirmed for their ability to stimulate a specific T cell response. To further filter which of these 8 sequence regions presented on the APC surface could be conferring specific T cell reactivity, peptides spanning these 8 sequence regions were synthesized. In addition, peptides spanning the rest of the CDR regions of AMG 212 were proactively synthesized alongside the MAPPS-identified sequence regions to completely account for AMG 212’s most novel sequence regions with the highest potential for immunogenicity.

**Figure 6:**
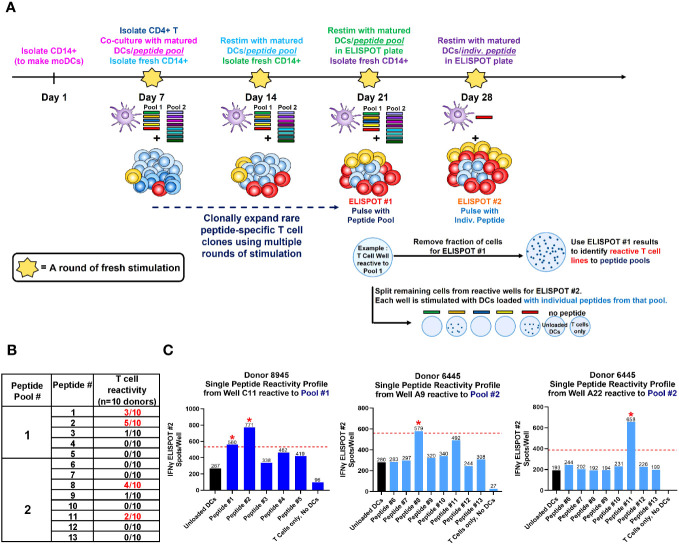
Restimulated T cell line assay on healthy donor PBMCs. A schema of the restimulated T cell assay is shown in **(A)**. To simulate an antigen-experienced memory response, isolated CD4+ T cells were stimulated with multiple rounds of autologous monocyte-derived DCs (moDCs) pulsed with our suspect peptides in a 4-week co-culture. In the week prior to each stimulation, CD14+ cells were isolated from healthy donor PBMC, differentiated into immature DCs with IL-4 and GM-CSF, then separately loaded with 5 µg/mL CEFTA peptide pool or 5 µM PADRE peptide (positive controls), or 5 µM of Peptide Pool #1 or #2 (test peptides) and matured with TNF-α and IL-1β for 48 hours. On Day 7, autologous CD4+ T cells were isolated and seeded at 2 X 10^5^/well and stimulated with peptide-loaded DC weekly for the next 21 days. Freshly-loaded and matured DCs were added to the T cell culture every 7 days, and the culture medium was refreshed every 7 days with IL-2 and IL-7. On Day 21, a fraction (4-5 X 10^4^) of CD4+ T cells were removed from each well and stimulated with peptide pool-loaded DCs in pre-coated Human Interferon-γ ELISPOT plates, visualized and counted for spots after a 48 hr incubation. On Day 28, peptide pool-specific T cell lines identified from ELISPOT #1 were then fractionated and stimulated with individual peptide-loaded DCs in pre-coated Human Interferon-γ ELISPOT plates, visualized and counted for spots 48 hr later as before. The table in **(B)** shows the aggregate incidence of individual peptide reactivity among the 10 donors tested, after performing this assay 3 times. The bar graphs in **(C)** show the individual peptide reactivity profile (as determined by ELISPOT #2) of Donor 8945 and Donor 6445. A T cell line was deemed reactive to an individual peptide if the spot counts were 2-fold higher than unloaded DC controls, with a minimal difference of 30 spots (above the cut-off value). The red dotted line represents the cut-off value in each plot for that T cell line, which may be different between wells based on the unloaded DC control. Reactive peptides are denoted with a red asterix *. The HLA allele subtypes of the 10 donors used in this assay are found in **Supplementary Table 2**.

To determine which of these suspect epitopes could confer T cell reactivity, a restimulated T cell line assay using healthy donor PBMCs was developed in-house, with modifications to what has been described previously (16, 17, 33). The restimulated T cell line assay is in essence, an extension of the traditional DC:T assay that evaluates sequence-based immunogenicity risk (32). A key differentiating factor is that the restimulated T cell line assay utilizes multiple rounds of stimulation instead of one. This serves two purposes. First, this approach recapitulates the chronic dosing regimen that AMG 212 patients experienced and therefore simulates an antigen-experienced memory response. Second, because naïve healthy donors were being used in this assay, multiple stimulations aid in increasing the rare antigen-specific precursor T cell clonal frequencies found in naive individuals (1:10^7^) to those found in memory responses (~1:10^3-5^) (34). The assay schema for the restimulated T cell line assay is shown in **Figure 6A**. The restimulated T cell line assay was performed 3 times, with a total of 10 donors. The HLA allele subtypes of these donors are shown in **Supplementary Table 2**.

The accrued assay results showed that of the 13 suspect peptides tested, 4 peptides showed T cell reactivity in more than 1 donor (**Figure 6B**). These 4 peptides were Peptide #1, 2, 8 and 11. Of the 10 donors, 3 donors were reactive to Peptide #1, 5 donors to Peptide #2, 4 donors to Peptide #8 and 2 donors to Peptide #11 (**Figure 6B**). In assays where the same donor was repeated, specific peptide reactivity could be reproduced. Representative ELISPOT images along with corresponding spot counts showing the individual peptide reactivity profile of 2 donors are shown in **Figure 6C**. In these examples, Donor 8945 was observed to react to Peptides #1 and 2 from Pool 1, while Donor 6445 was observed to react to Peptides #8 and 11 from Pool 2 (**Figure 6C**). Notably, peptides such as peptide #4-7, 9-10, 12 and our self-tolerant peptide #13 negative control, did not show T cell reactivity consistently across assays. Peptide #4 and #5, despite being presented by 7 and 8 out of 20 donors respectively in the MAPPS assay (**Figure 5B**), failed to confer T cell reactivity in the restimulated T cell line assay. MAPPS can be under-predictive if the appropriate sensitivity is not applied (12, 32). However, this was not the case in our MAPPS assays, as we had sufficient consistency and sensitivity across both rounds of MAPPS assays (see Methods).The results from the restimulated T cell line assay align with our expectations that only a subset of sequences identified from the MAPPS assay, translate into T cell reactivity.

With our top suspect sequence regions in hand, we sought to ascertain which of these sequences could be driving AMG 212 immunogenicity in a clinical memory recall assay (19) using patient PBMCs. The recall assay capitalizes on an antigen-experienced memory response from ADA+ subjects, in which the patient’s peptide-specific T cell clonal frequency has been expanded. In this assay, upon *ex vivo* stimulation from the immunogenic peptide(s), this pool of peptide-specific memory T cell clones within ADA+ patient PBMCs can be recalled upon to secrete Interferon-γ, detectable by ELISPOT.

Ideally, we would have performed this recall assay using PBMCs from AMG 212 ADA+ patients. However, at this point in our investigation, the AMG 212 FIH clinical trial had already concluded, and we were unable to obtain PBMCs from AMG 212 ADA+ subjects retrospectively. However, a follow-on molecule to AMG 212, AMG 160 (half-life extended BiTE^®^ molecule), was being investigated in a FIH trial at that time (ClinicalTrials.gov, NCT03792841).

AMG 212 and AMG 160 were observed to have 98.4% sequence identity. Importantly, comparing our top peptide sequence suspects #1, 2, 8, 8.5 and 11 in AMG 212 to analogous regions in AMG 160, we found Peptide #1, 2, 8 and 8.5 to be identical. Peptide #11 was 2 amino acids different compared to the analogous sequence in AMG 160. To determine if this difference could affect HLA class II binding, we utilized structural modeling to determine where the critical nonamer binding core could exist within that sequence. The results of these modeling efforts predicted that those 2 amino acid positions were not anchor residues for HLA class II binding. Therefore, Peptide #11 would likely be bound to HLA class II similarly to the analogous AMG 160 peptide sequence, and hence recognized similarly by AMG 160 patients’ T cell clones.

Unlike AMG 212, which was administered by continuous IV infusion, AMG 160 was administered by short-term IV infusion over 1 hour, every 2 weeks, after the target dose was reached. Yet, despite being administered intravenously, AMG 160 engendered clinically significant immunogenicity. As of Sep 19^th^ 2020 (an earlier data-cut), as disclosed in the virtual ESMO 2020 presentation describing interim results of the AMG 160 FIH study, 6 of 30 (20.0%) patients evaluated developed ADAs which affected drug exposure between cycles 1 and 10 (35). The full AMG 160 ADA dataset, which evaluated a greater number of subjects, will be disclosed in an upcoming publication (in preparation). By comparison, the clinically meaningful AMG 160 ADA incidence was not as high as that of AMG 212, to which TE-ADA developed in 30/31 subjects (96.7%) within the first 6 cycles upon SC administration of AMG 212 (**Table 1.1**; **Figure 1A**).

Due to the high sequence identity, we postulated that the sequences driving immunogenicity to AMG 212 and AMG 160 were most likely the same. In addition, the disease population treated was comparable in the AMG 212 and AMG 160 FIH trials, further supporting the rationale to test our suspect AMG 212 peptide sequences using AMG 160 patient samples. We obtained End of Treatment (EOT) PBMC samples from patients in the AMG 160 FIH trial, to evaluate Peptide #1, 2, 8, 8.5 and 11 in the clinical memory recall assay. In total, PBMC samples from 9 ADA-positive and 8 ADA-negative patients from the AMG 160 FIH trial were assessed in this assay.

**Figure 7:**
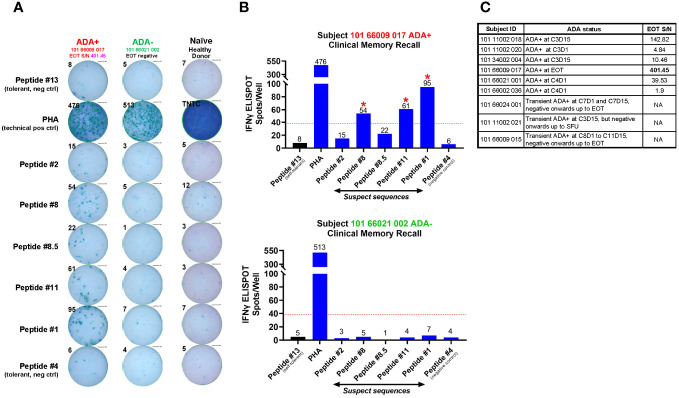
Clinical memory recall assay on patient PBMCs obtained at EOT from the AMG 160 FIH trial. Regardless of ADA status, 10 ml of whole blood was collected at the end of treatment (EOT) timepoint from patients enrolled in the AMG 160 First-in-Human (FIH) trial, Study 20180101. Whole blood was sent ambient to the central lab for same-day processing into PBMCs and stored frozen. Freshly thawed patient PBMCs were plated at 2 X 10^5^ cells per well, pulsed with individual peptides at 5 µM for 72 hr, and evaluated for a recall response via IFNγ ELISPOT. 10 ng/ml of GM-CSF was provided in the culture. Peptides # 1, 2, 8, 8.5 and 11 were experimental “suspect” sequences. Peptides #13 and #4 were negative control sequences that did not confer T cell reactivity, which we established previously from the restimulated T cell line assays. PBMC from an AMG 160-naive healthy donor was used as an additional negative control. Phytohemagglutinin (PHA) was used as a non-specific T cell activator and acted as a technical positive control for the ELISPOT assay. TNTC refers to “Too-numerous-to-count”. The ELISPOT image enumerating the IFNγ spot counts in response to *ex vivo* stimulation from these individual peptides is shown for ADA-positive subject 101 66009 017 and ADA-negative subject 101 66021 002 **(A)**, and depicted as bar plots in **(B)**. The red dotted line in the bar graphs represents the cut-off value calculated as a number with two-fold more spots in the presence of that individual peptide compared to self-tolerant Peptide #13 within the same subject, with a minimal difference of 30 spots. Peptides producing spot counts above the cut-off value were considered able to promote a recall response in these patient PBMCs (denoted by red asterix *). In total, PBMC samples from 9 ADA-positive and 8 ADA-negative patients from the AMG 160 FIH trial were assessed in this assay. The 9 ADA-positive subjects, their binding ADA onset and the magnitude (Signal-to-Noise, S/N) of ADA response at EOT (if found positive at EOT), are shown in the table in **(C)**.

Of the suspect sequences tested, Peptide #1, 8 and 11, but not #2 or 8.5, exhibited a recall response in a single AMG 160 ADA+ subject (**Figures 7A, B**). This was not observed in AMG 160 ADA-negative subjects or in AMG 160-naive healthy donor controls (**Figures 7A, B**). Notably, this subject with detectable peptide reactivity, had the highest magnitude of ADA at the EOT timepoint among our 9 ADA-positive subjects (**Figure 7C**). Five other ADA-positive subjects had comparatively lower magnitude (1-2 logs lower) of ADA response at the EOT timepoint, while the remaining 3 ADA-positive subjects had a transient ADA response and was found ADA-negative at the EOT timepoint (**Figure 7C**). Subsequent discontinuation of the AMG 160 FIH study precluded our ability to obtain more ADA+ patient PBMCs and perform additional recall assays.

Taken together, our multi-assay approach sequentially filtering potential epitopes starting from the level of MHC class II binding to recalling a clinical memory ADA response *ex vivo*, yielded at least 3 possible non-tolerant sequence-based epitopes in AMG 212.

In conclusion, the totality of data from our root cause investigation supports our hypothesis explaining the disparate TE-ADA incidence between the AMG 212 SC and CIV cohorts. The unfavorable combination of a subcutaneous drug delivery, in which a >20 kD protein such as AMG 212 would have had to traffic through secondary lymphoid tissue first, together with at least three non-tolerant T cell epitopes present within the AMG 212 sequence, most likely contributed to the sustained, high-titer ADA response to AMG 212.

## Discussion

The emergence of clinically impactful immunogenicity during development is potentially detrimental to patients from two standpoints. First, if the ADAs are associated with certain adverse events. Second, if the ADAs are neutralizing and/or significantly reduce exposure, this may prevent any efficacy response or curtail durability of response. The AMG 212 FIH study’s SC arm was an unfortunate case-in-point illustrating the latter in an oncology indication. The decision to switch route of administration from SC to CIV mid-study enabled signs of drug activity to be observed, likely in part because ADAs did not develop in the CIV arm and exposure was sustained. Remarkably, in two CIV subjects, durable PSA responses lasting more than one year were achieved.

The root cause of the immunogenicity observed in the SC arm was initially attributed to the route of administration and treatment regimen. Preclinical and clinical data, including internal Amgen clinical data, support our current understanding that IV administration, in general, has a lower immunogenicity risk than SC administration (36–39). However, there are several instances where there are no differences in immunogenicity rates to the same biologic administered SC and IV, such as in the case of ACTEMRA® (tocilizumab) and ORENCIA® (abatacept) (40, 41).

In certain cases, a lower dose administered intermittently may be more immunogenic than a larger dose administered without interruption (42). Due to the very short half-life (2-3 hr) and fast clearance of AMG 212, a more frequent dosing (once daily dosing) was necessitated to preserve exposure in subjects receiving AMG 212 by the SC route. In contrast, for the CIV dose administrations, subjects received AMG 212 as a continuous IV infusion at a constant flow rate given over 5 consecutive weeks followed by a treatment-free interval of 1 week.

When a foreign protein such as AMG 212 was injected subcutaneously at microgram levels, the frequent daily administration in this setting may have elicited ADA formation due to repeated boosting. Thus, the combination of low-dose, high-frequency and historically more immunogenic route of administration may have elicited the ADA response in the SC arm. As the SC portion of the study was terminated early, strategies such as optimizing dosing frequency to mitigate ADA development was not attempted.

Apart from the dose and dosing frequency, we considered aspects of SC drug delivery that could influence the induction of an ADA response. Drug delivery via the SC route relies on uptake from the interstitial domain of the subcutis. It is well-established that the molecular size of proteins injected SC determines their fate and path to the systemic circulation (28, 29). They have two potential routes for uptake and biodistribution. Lower molecular weight drugs (<20 kD), including small molecules such as insulin, can enter the general circulation directly through blood capillaries. However, higher molecular weight (>20kD) drugs, which include BiTE^®^ molecules such as AMG 212, must traffic through the interstitial matrix of the subcutis to the peripheral lymphatic system first, before entering the systemic circulation (28, 29).

Thus, SC-delivered AMG 212 would have encountered APCs and other immune cells through a series of lymph nodes enroute to the thoracic duct, before reaching the peripheral circulation. This likely provided more opportunity and time for APCs to phagocytose the drug and engender an immune response to AMG 212. CIV-delivered AMG 212 however, directly entered the bloodstream from inception, bypassing the peripheral lymphatic system on the first pass through the body. However, relying on this explanation alone would be an oversimplification, as not all SC-injected protein therapeutics above 20kD in size elicit ADA responses. Conversely, IV-injected protein therapeutics still run the risk of engendering clinically meaningful immunogenicity, as we observed in the case of AMG 160.

Other factors associated with the anatomy of the skin were considered as well. The rapid egress of the drug product into the skin, an organ containing a high frequency of APCs (43), together with a possible depot effect where the drug product forms or stays in aggregates in the interstitial SC space (44, 45) compared to dispersal in high-flow, fluid-rich IV environment, were all plausible reasons why the immunogenic response was triggered in SC-administered patients. However, despite intense topical GC treatment at the injection site to suppress local APC response, this mitigation strategy proved unsuccessful. This suggested that the induction of immunogenicity to SC-delivered AMG 212, was not skin-deep and belied a different and/or further root cause.

Apart from the route of administration, the causes of an immunogenic response to therapeutic proteins include patient-related factors, such as genetic background, baseline immunologic status from either disease state or concomitant medications, and product-related factors, such as attributes incurred during manufacture of the drug and the amino acid sequence of the drug itself. In this report, we investigated as many of these potentially contributing factors as we were able.

As the patients’ prior lines of therapies and disease indication were comparable between the patients enrolled into the SC and CIV arms, such patient-specific factors were first ruled out. Baseline immune status (**Figure 4**) and product quality attributes such as HMWS in the GMP lots (**Table 2**) were comparable between the SC and CIV arms, thus ruling these factors out as well. We therefore focused our efforts on intrinsic factors of the drug, such as structure-based B cell epitopes and more importantly, sequence-based, T cell epitope(s) that may explain the immunogenicity to AMG 212.

Identification of immunogenic epitopes in biologics is not without precedent, and several groups have recently successfully done so for T cell epitopes (17, 19) and even B cell epitopes (46). Through a series of *in vitro* assays including MAPPS, restimulated T cell line and clinical memory recall assays, we identified at least 3 possible sequence drivers of AMG 212 immunogenicity (**Figures 5**–**7**).

While considered the gold standard, obtaining patient PBMC samples for the clinical memory recall assay proved a unique challenge because the AMG 160 FIH study was nearing conclusion by the time we introduced this novel PBMC sample collection for the purposes of performing the recall assay. A limited number of AMG 160 patient PBMC samples were ultimately collected to evaluate the suspect sequence regions. Low cell viability in the patient PBMCs precluded our ability to perform high throughput analyses evaluating more sequences.

Furthermore, we observed that the recall assay was successful in detecting a memory response only in an ADA+ subject with a robust magnitude of ADA at the time of PBMC collection. This is presumably due to an ongoing high-affinity antibody response, in which expanding CD4+ T cell clones continue to provide classical help via the CD40-CD40L axis to perpetuate the antibody response. The ability to detect recall responses may therefore be largely dependent on the strength of the ADA response at the point of PBMC sample collection, which is variable and unpredictable in the clinic. These factors should be carefully deliberated upon when seeking out culprit T cell epitopes responsible for clinical immunogenicity using recall assays.

Both the MAPPS and restimulated T cell assays utilized healthy donor cells. This may not recapitulate the diseased condition where differential proteasomal processing of antigens and post-translational modifications of the epitopes may be taking place. Therefore, sequences showing T cell reactivity in assays using cells derived from healthy donor PBMC, may not fully replicate the epitopes driving a clinical ADA response to the same biologic in a disease setting. Even in a clinical memory recall assay that utilizes patient PBMCs, the number of possible suspect sequences that can be tested is ultimately limited by the PBMC viability and numbers collected in the patient sample. In addition, although this report focused largely on finding epitopes driving a T-dependent ADA response, a potential role of T-independent B cell responses driving AMG 212 immunogenicity cannot be excluded.

In this manuscript, we disclosed the amino acid residue numbering of the suspect sequence regions but not the amino acid sequences as the latter represent proprietary information. However, not disclosing the actual sequences themselves does not compromise the interpretations and conclusions of this report. In general, ADAs have the greatest potential to develop in response to antigen-specific sequences in the CDRs of even fully-human antibodies as they are deemed the most foreign part of the biologic (6). Indeed, 2 of the 3 identified non-tolerant epitopes spanned the CDR regions of AMG 212. This result was not unexpected. However, some outstanding questions remain.

Of the 3 non-tolerant epitopes identified, was one more immunodominant than the other two? Were there more epitopes we would have identified had we been able to perform more recall assays? AMG 160 differs structurally from AMG 212 as it has an additional “add-on” of a single chain Fc on the C-terminus end of the CD3 binder, for the purposes of half-life extension. With the additional Fc portion on AMG 160, could this key structural difference, which would inherently generate different overall B cell epitopes, result in different ADA responses to AMG 212 and AMG 160? The additional Fc portion could also result in differential antigen uptake and proteasomal processing of AMG 160 in APCs. Could epitopes have been missed in using AMG 160 subject PBMCs, instead of AMG 212 subject PBMCs? If there were intrinsic immunogenic regions within the AMG 212 amino acid sequence itself that was likely driving the ADA responses, why were ADAs not observed in the CIV arm as well? Could the continuous IV administration have induced immune tolerance over time to AMG 212, permitting AMG 212 to go “unseen” by the immune system?

Another potential explanation for the lack of ADAs in the CIV arm pertains to the dose levels administered. The maximum tolerated dose was not reached before the study was discontinued (not due to lack of efficacy or safety reasons). Anecdotally, across several T cell engager trials, we have observed that intra-subject dose-escalation can sometimes result in *de novo* development of ADAs. This has been observed even in situations where a patient had been ADA-negative for a significant amount of time prior to the intra-subject dose-escalation. In the AMG 212 FIH study, it is possible that had dose escalation in the CIV arm been pursued, clinically meaningful ADAs may have been detected at higher dose levels. However, as we did not continue further dose escalation past 80 µg/d, we acknowledge that this remains mere speculation.

**Figure 8:**
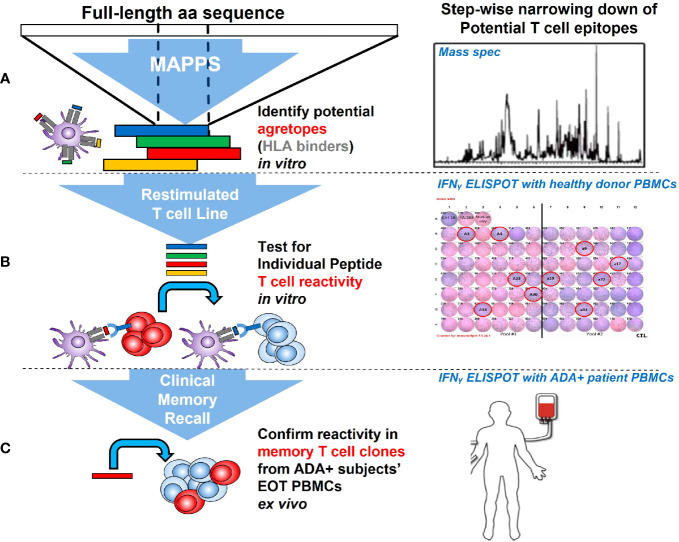
Stepwise approach used to identify sequence-based T cell epitopes driving AMG 212 immunogenicity. Starting at the level of APC recognition and presentation, MHC class II-associated peptide proteomics (MAPPS) was used to parse out sequence regions that were naturally processed and presented by HLA class II on the APC surface **(A)**. To further narrow down suspect sequences, peptides representing the MAPPS-identified sequence regions and all other CDR regions were synthesized and tested for individual peptide reactivity in a restimulated T cell line assay, which recapitulates an antigen-specific memory response in healthy donors **(B)**. Peptides that conferred T cell reactivity through this assay were then tested in a clinical memory recall assay, to confirm the peptide’s ability to produce a recall response in patients who have developed a robust anti-drug antibody response in the clinic **(C)**.

This body of work, built upon many others, establishes a thought process and a systematic approach in addressing how a sponsor may identify culprit T cell epitopes driving clinical immunogenicity (**Figure 8**). Upon their identification, culprit T cell epitopes can be removed or de-immunized in the next iteration of the biologic. However, such re-engineering efforts face an arguably uphill task. Although it has been done previously (47–50), de-immunizing key amino acid residues requires extensive modeling to determine the nonamer cores (51) within the identified suspect sequences. Unlike the closed binding pocket of MHC class I, the MHC class II binding pocket is open and more flexible (52). Within the nonamer cores, determining anchor residues in the binding pocket or those protruding into the TCR for possible replacement, would be key to de-immunization. Importantly, while disruption of HLA class II binding would be the goal of these modeling efforts, these point mutational analyses must fulfill other pertinent, non-trivial criteria. These include ensuring that upon modifying the CDRs, the binding affinity and potency of the target binders are not affected, and that the overall antibody construct remains stable and intact, such that the drug retains its intended functionality.

When a biologic exhibits high sequence-based risk based on available prediction tools, downstream assays to confirm possible epitopes should be initiated. One can envision that the tiered approach we utilized to identify culprit epitopes retrospectively, can also be implemented prospectively, to de-risk molecules before they enter the clinic. Indeed, others have built mechanistic models that additionally account for the drug’s mode of action when attempting to predict a molecule’s clinical immunogenic risk (53). Clearly, there exists a concerted effort from industry and regulators alike to shift from viewing ADA development as an aleatory risk to an informed one.

Clinical immunogenicity may still accompany development of immunomodulatory drugs despite best efforts in predicting immunogenic risk and de-immunizing as much as possible upfront. Biologics whose mode of action potently ablates B cells or inhibits their maturation and differentiation, discernibly run a much lower risk of developing clinically meaningful ADAs, even when administered in the SC setting. Notably, the first approved T cell engager worldwide, BLINCYTO^®^ (blinatumomab) (CD19-targeting), although approved as a CIV formulation, has since been tested as a SC formulation in both Relapsed/Refractory indolent Non-Hodgkin’s Lymphoma (NHL) and Acute Lymphoblastic Leukemia (ALL). In both of these trials, anti-blinatumomab antibodies were not detected (54, 55).

In the absence of early-onset, clinically impactful, “showstopping” ADAs, sponsors and regulatory agencies alike may consider raising their tolerance threshold to ADAs for biologics exhibiting a favorable risk: benefit ratio, and in which the ADA impact to clinical response is none, unclear or unknown.

The recent approval of KIMMTRAK^®^ (tebentafusp-tebn), a first-in-class T cell engager for HLA-A 02:01-positive metastatic uveal melanoma patients may be a case-in-point. A 29-33% binding ADA incidence graces the label of KIMMTRAK^®^. High-titer ADA was shown to decrease exposure by 97% (56). However, the ADAs did not appear to impact overall survival. Such approvals suggest that an increased tolerance of biologics with significant ADA in light of a favorable risk: benefit ratio may already be underway.

Mitigation of ADAs with a variety of strategies during early clinical development have been considered over the years for different disease indications (57). This may be feasible in a disease population where the mitigation strategy is part of standard of care. However, such added interventions are generally not feasible in an already heavily pre-treated oncology population, and where other weakly or non-immunogenic therapies may be available as alternatives. In an age where immunomodulatory drugs dominate oncology pipelines across industry, we propose that clinical monitoring of immunogenicity for this class of drugs in early phase trials is no longer obligatory, but an imperative for onward progress to pivotal stage development.

## Data availability statement

The original contributions presented in the study are included in the article/**Supplementary Material**. Further inquiries can be directed to the corresponding author.

## Ethics statement

The studies involving humans were approved by (1) Krankenhaus der Barmherzigen Schwestern Linz, Ethikkommission der Barmherzigen Schwestern Linz, Seilerstätte 4, 4010 Linz, Austria (2); Ethikkommission des Landes Oberösterreich, Kepler Universitätsklinikum Neuromed Campus, Wagner-Jauregg Weg 15, 4020 Linz, Austria (3); Universitätsklinikum Heidelberg, Ethikkommission der Med. Fakultät Heidelberg, Alte Glockengießerei 11/1, 69115 Heidelberg, Germany (4); Klinikum der Universität Würzburg, Ethik-Kommission bei der Medizinischen Fakultät, Institut für Pharmakologie und Toxikologie, Versbach Str. 9, 97078 Würzburg, Germany (5); Landesamt für Gesundheit und Soziales, Geschäftsstelle der Ethik-Kommission des Landes Berlin, Fehrbelliner Platz 1 (Dienstgebäude), 10707 Berlin, Germany. The studies were conducted in accordance with the local legislation and institutional requirements. The participants provided their written informed consent to participate in this study. Written informed consent was obtained from the individual(s) for the publication of any potentially identifiable images or data included in this article.

## Author contributions

HP: Conceptualization, Data curation, Formal Analysis, Investigation, Methodology, Writing – original draft, Writing – review & editing. KH: Formal Analysis, Methodology, Writing – review & editing. NT: Formal Analysis, Methodology, Writing – review & editing. BW: Writing – review & editing, Methodology. CB: Formal Analysis, Methodology, Writing – review & editing. CW: Formal Analysis, Methodology, Writing – review & editing. MM: Formal Analysis, Methodology, Writing – review & editing. SW-R: Conceptualization, Formal Analysis, Funding acquisition, Writing – review & editing. GK: Conceptualization, Formal Analysis, Funding acquisition, Writing – review & editing. SS: Conceptualization, Data curation, Formal Analysis, Investigation, Methodology, Project administration, Supervision, Writing – review & editing. RB: Conceptualization, Investigation, Writing – review & editing. H-DH: Conceptualization, Investigation, Writing – review & editing. WL: Conceptualization, Investigation, Writing – review & editing. CG: Conceptualization, Investigation, Writing – review & editing. TE: Data curation, Formal Analysis, Investigation, Methodology, Project administration, Supervision, Writing – review & editing. BT: Investigation, Writing – review & editing. DM: Formal Analysis, Funding acquisition, Methodology, Project administration, Supervision, Writing – original draft, Writing – review & editing, Conceptualization, Data curation.

## Supplementary material

Supplementary Figure 1 Dosing schema and ADA sampling timepoints of the subcutaneous (SC) **(A)** and continuous IV (CIV) infusion **(B)** arms of the AMG 212 First-in-Human clinical study. Cycles are depicted by green arrows; dosing schedules depicted by blue font and ADA sampling timepoints depicted by red arrows. The terms “C” refers to cycle, “D” refers to day and “EOIP” refers to End-of-Investigational Product. In the SC arm, AMG 212 was administered daily by SC injection, with no breaks between cycles **(A)**. In the SC arm, ADA samples were collected predose on Cycle 1 Day 1, 8 and 15, on Day 1 of each cycle from Cycle 2 to 8, on Day 1 of every second cycle thereafter and at least 36 hr after the last dose of AMG 212 **(A)**. In the CIV arm, AMG 212 was administered as a continuous IV infusion, using an on-body portable infusion pump and central venous port system. In the first 4 cycles (first 12 weeks on study), patients received treatment on a “5 week on-1 week off” schedule, whereby AMG 212 was administered over 5 weeks, followed by a treatment-free interval of 1 week. From cycle 5 onwards, patients could continue treatment on the “5 week on-1 week off” schedule or switch to a “4 week on-2 week off” schedule, at the discretion of the investigator and the subject **(B)**. In the CIV arm, ADA samples were collected predose on Cycle 1 Day 1, 8 and 15, on Day 1 of each subsequent cycle and at least 36 hr after the last dose of AMG 212 **(B)**.

Supplementary Figure 2 Comparison of AMG 212 concentration (ng/mL, y-axis, log-10 scale) from SC-dosed subjects receiving 72 µg/day (top), 144 µg/day (middle) and 172 μg/day (bottom) by ADA positivity status per subject with available PK data **(A)**; ADA status, exposure impact and PSA response correlation analyses using 2X2 tables **(B, C)**. The 1-hour post-dose concentration (x-axis) for cycle 1 day 1 (C1D1HR1), day 15 (C1D15HR1), cycle 2 day 1 (C2D1HR1), cycle 3 day 1 (C3D1HR1) and cycle 4 day 1 (C4D1HR1) are shown for comparison. Negative ADA status is shown in gray and positive ADA status is shown in dark red. All subjects with available data from the subcutaneous cohort including subjects with co-administration of glucocorticoid treatment are included. Samples below the lower limit of quantitation (LLOQ) were assigned 0.15 ng/mL **(A)**. To determine the correlation between ADA status and exposure impact (PK < LLOQ at or after ADA onset), the 2X2 table shown in **(B)** was utilized and a logistic regression model applied. The results show that the odds ratio is 0.080 (95% CI: <0.001, 8.698), *p-value=0.2915*. While the numbers in the table show a trend of exposure impact in ADA-positive subjects, due to the small sample size, this trend is not statistically significant. To determine the correlation of Exposure Impact with PSA rebound, the 2X2 table shown in **(C)** was utilized and a logistic regression model applied. The results show that the odds ratio is 25.998 (95% CI: 1.118, 604.431), *p-value=0.0424*. The numbers in the table show a clear trend of PSA rebound in exposure-impacted subjects, with a p-value reaching significance (<0.05). Due to the limited sample size of these correlation analyses, these analyses are presented herein in a descriptive manner.

Supplementary Figure 3 Peripheral blood CD14+ monocyte counts over time between patients who did or did not receive topical glucorticosteroids (GC) at the 144 μg/d **(A)** and 172 µg/d **(B)** dose levels in the SC arm of the AMG 212 clinical study. Monocyte counts were tabulated over time, before and during AMG 212 SC dosing. “C” refers to cycle and “D” refers to day. “PRE” refers to predose. Each symbol/connecting line represents individual subjects who did apply topical GC (+GC) or did not (-GC) on their injection sites. The apparent decrease in monocyte count from Day -7 to Cycle 1 Day 1, during which clobetasol propionate is administered, may be confounded by the prophylactic Dexamethasone to mitigate against Cytokine Release Syndrome (CRS) before the start of dosing in these subjects. To determine whether GC impacted peripheral blood monocyte counts over time, an unpaired t test was applied to the data comparing both groups at each time point. At the 144 ug/d cohort, monocyte counts of +GC (n=3) compared to -GC (n=3) subjects showed a *p-value* of 0.28, 0.15 and 0.44 at the screening, Cycle 1 Day 1 (C1D1) predose and Cycle 1 Day 15 (C1D15) predose timepoints respectively. At the 172 ug/d cohort, monocyte counts of +GC (n=6) compared to -GC (n=3) subjects showed a *p-value* of 0.43, 0.15 and 0.99 at the screening, C1D1 predose and C1D15 predose timepoints respectively. Collectively, the data show that the use of topical GC did not significantly change peripheral blood monocyte counts.

Supplementary Table 1 HLA subtypes.

Supplementary Table 2 HLA subtypes.
