# Supplementary material for: Characterization and root cause analysis of immunogenicity to pasotuxizumab (AMG 212), a prostate-specific membrane antigen-targeting bispecific T-cell engager therapy
Source: Front Immunol. 2023 Oct 23;14:1261070. doi: 10.3389/fimmu.2023.1261070 (PMC10628759; doi:10.3389/fimmu.2023.1261070)
Supplement: Supplementary Figure 1 — Dosing schema and ADA sampling timepoints of the subcutaneous (SC) (A) and continuous IV (CIV) infusion (B) arms of the AMG 212 First-in-Human clinical study. Cycles are depicted by green arrows; dosing schedules depicted by blue font and ADA sampling timepoints depicted by red arrows. The terms “C” refers to cycle, “D” refers to day and “EOIP” refers to End-of-Investigational Product. In the SC arm, AMG 212 was administered daily by SC injection, with no breaks between cycles (A). In the SC arm, ADA samples were collected predose on Cycle 1 Day 1, 8 and 15, on Day 1 of each cycle from Cycle 2 to 8, on Day 1 of every second cycle thereafter and at least 36 hr after the last dose of AMG 212 (A). In the CIV arm, AMG 212 was administered as a continuous IV infusion, using an on-body portable infusion pump and central venous port system. In the first 4 cycles (first 12 weeks on study), patients received treatment on a “5 week on-1 week off” schedule, whereby AMG 212 was administered over 5 weeks, followed by a treatment-free interval of 1 week. From cycle 5 onwards, patients could continue treatment on the “5 week on-1 week off” schedule or switch to a “4 week on-2 week off” schedule, at the discretion of the investigator and the subject (B). In the CIV arm, ADA samples were collected predose on Cycle 1 Day 1, 8 and 15, on Day 1 of each subsequent cycle and at least 36 hr after the last dose of AMG 212 (B). [file DataSheet_1.docx]

**A**

**
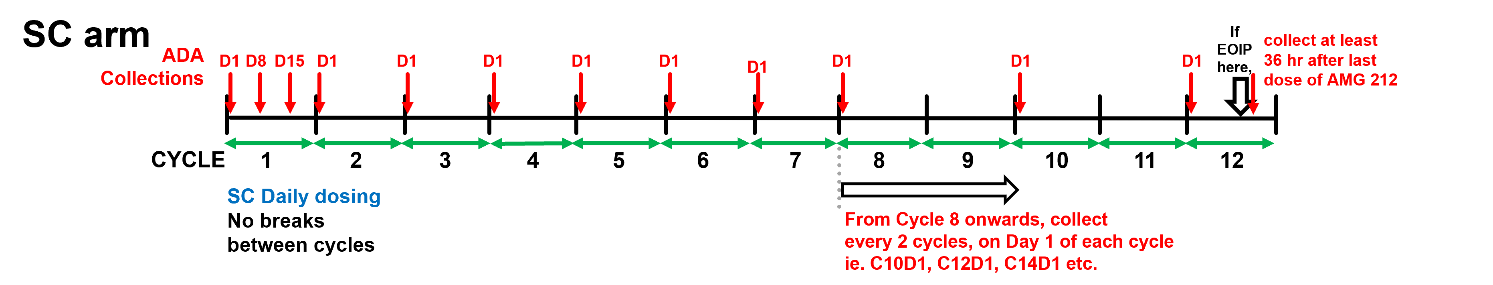
**

**B**

**
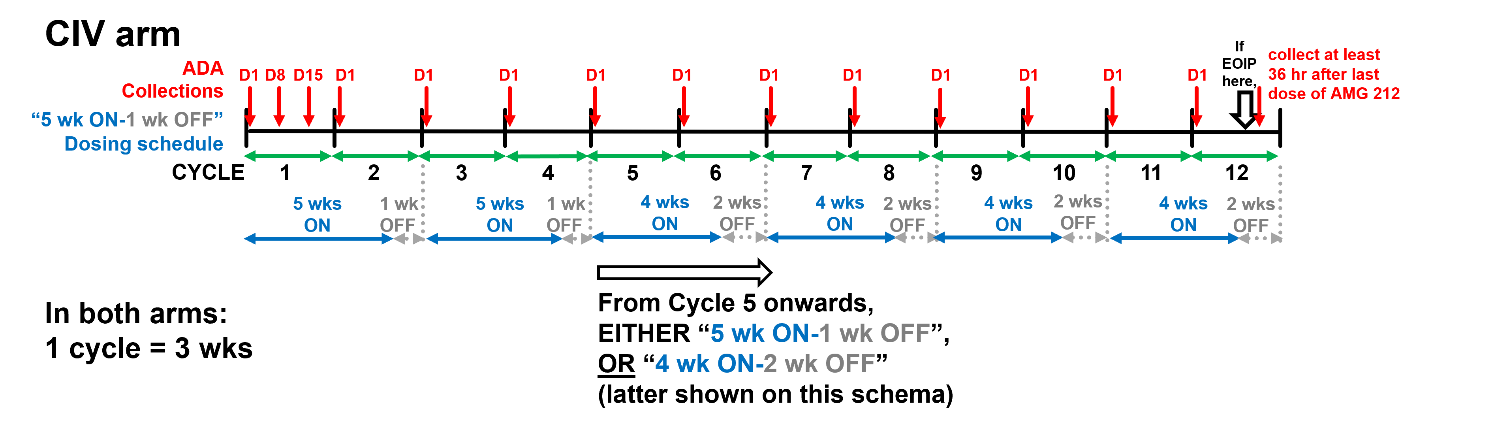
**

**Supplementary Figure 1.** Dosing schema and ADA sampling timepoints of the subcutaneous (SC) **(A)** and continuous IV (CIV) infusion **(B)** arms of the AMG 212 First-in-Human clinical study. Cycles are depicted by green arrows; dosing schedules depicted by blue font and ADA sampling timepoints depicted by red arrows. The terms “C” refers to cycle, “D” refers to day and “EOIP” refers to End-of-Investigational Product. In the SC arm, AMG 212 was administered daily by SC injection, with no breaks between cycles **(A)**. In the SC arm, ADA samples were collected predose on Cycle 1 Day 1, 8 and 15, on Day 1 of each cycle from Cycle 2 to 8, on Day 1 of every second cycle thereafter and at least 36 hr after the last dose of AMG 212 (**A**). In the CIV arm, AMG 212 was administered as a continuous IV infusion, using an on-body portable infusion pump and central venous port system. In the first 4 cycles (first 12 weeks on study), patients received treatment on a “5 week on-1 week off” schedule, whereby AMG 212 was administered over 5 weeks, followed by a treatment-free interval of 1 week. From cycle 5 onwards, patients could continue treatment on the “5 week on-1 week off” schedule or switch to a “4 week on-2 week off” schedule, at the discretion of the investigator and the subject **(B)**. In the CIV arm, ADA samples were collected predose on Cycle 1 Day 1, 8 and 15, on Day 1 of each subsequent cycle and at least 36 hr after the last dose of AMG 212 **(B)**.

**
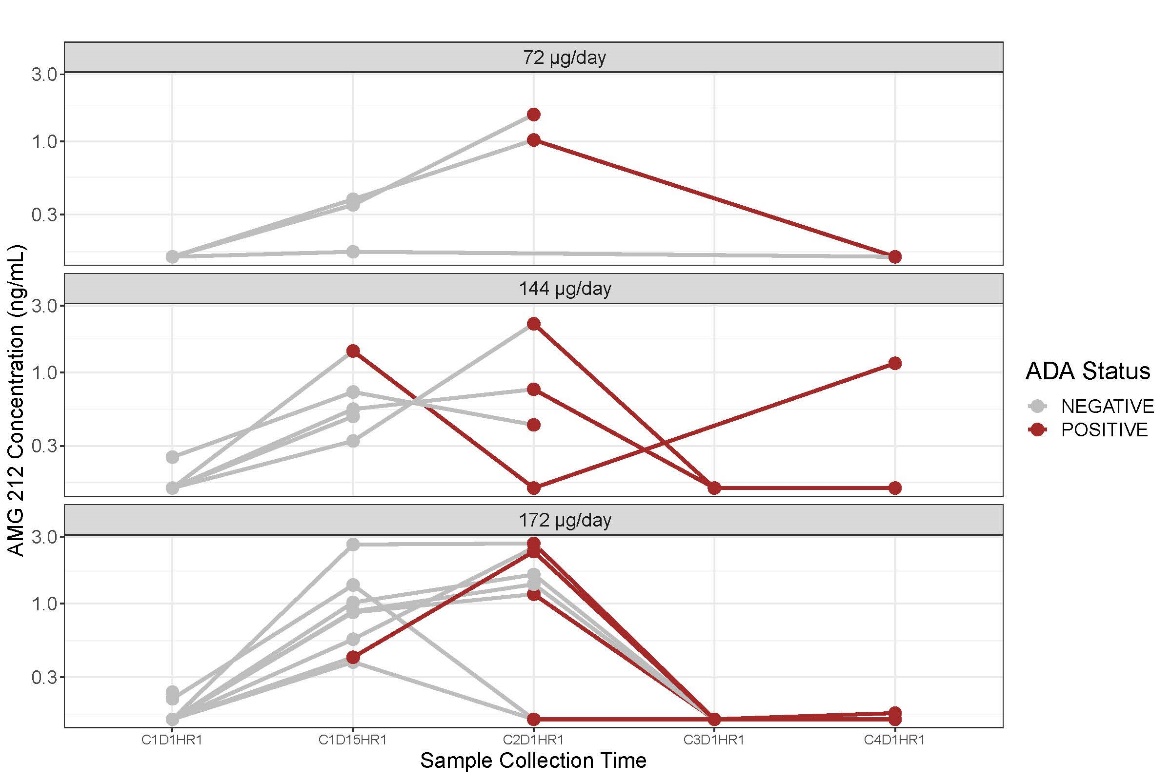
**

**A**

**B**

|  | Exposure impact/ YES | Exposure impact/ NO |
| --- | --- | --- |
| ADA-positive | 14 | 3 |
| ADA-negative | 0 | 1 |

**C**

|  | Exposure impact/ YES | Exposure impact/ NO |
| --- | --- | --- |
| PSA rebound/YES | 13 | 1 |
| PSA rebound/NO | 1 | 2 |

**Supplementary Figure 2.** Comparison of AMG 212 concentration (ng/mL, y-axis, log-10 scale) from SC-dosed subjects receiving 72 µg/day (top), 144 µg/day (middle) and 172 µg/day (bottom) by ADA positivity status per subject with available PK data (**A**); ADA status, exposure impact and PSA response correlation analyses using 2X2 tables (**B, C**). The 1-hour post-dose concentration (x-axis) for cycle 1 day 1 (C1D1HR1), day 15 (C1D15HR1), cycle 2 day 1 (C2D1HR1), cycle 3 day 1 (C3D1HR1) and cycle 4 day 1 (C4D1HR1) are shown for comparison. Negative ADA status is shown in gray and positive ADA status is shown in dark red. All subjects with available data from the subcutaneous cohort including subjects with co-administration of glucocorticoid treatment are included. Samples below the lower limit of quantitation (LLOQ) were assigned 0.15 ng/mL (**A**). To determine the correlation between ADA status and exposure impact (PK < LLOQ at or after ADA onset), the 2X2 table shown in (**B**) was utilized and a logistic regression model applied. The results show that the odds ratio is 0.080 (95% CI: <0.001, 8.698), *p-value=0.2915*. While the numbers in the table show a trend of exposure impact in ADA-positive subjects, due to the small sample size, this trend is not statistically significant. To determine the correlation of Exposure Impact with PSA rebound, the 2X2 table shown in (**C**) was utilized and a logistic regression model applied. The results show that the odds ratio is 25.998 (95% CI: 1.118, 604.431), *p-value=0.0424*. The numbers in the table show a clear trend of PSA rebound in exposure-impacted subjects, with a *p-value* reaching significance (<0.05). Due to the limited sample size of these correlation analyses, these analyses are presented herein in a descriptive manner.

**A B**


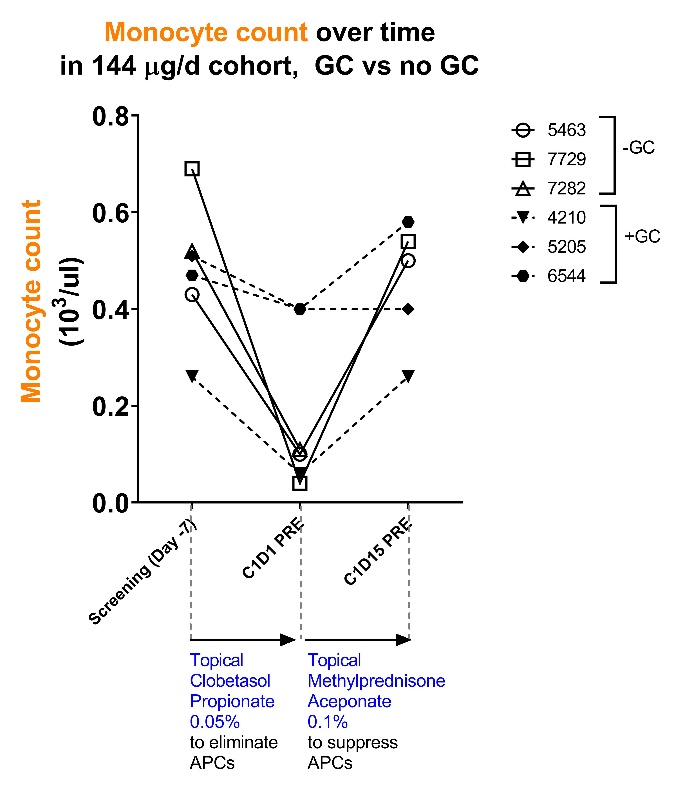

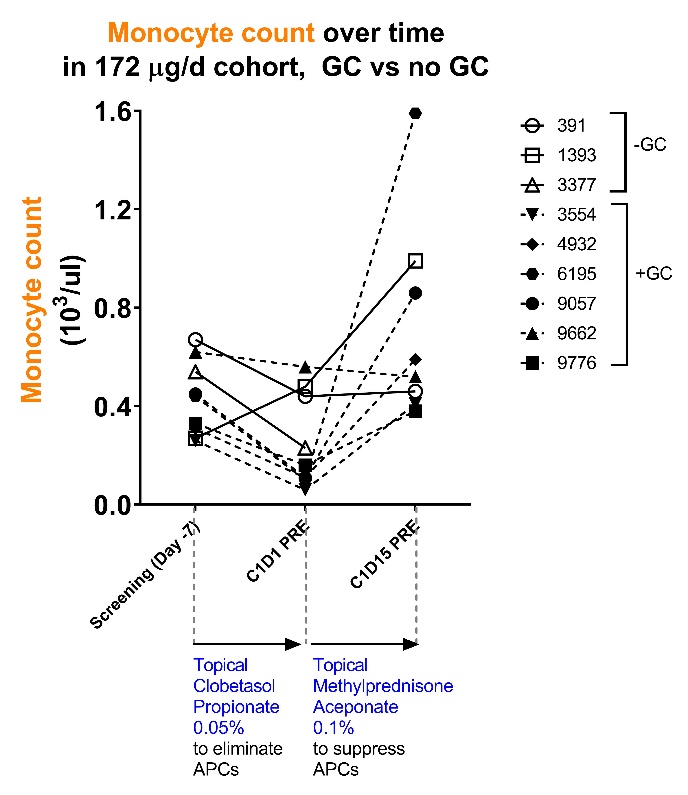


**Supplementary Figure 3.** Peripheral blood CD14+ monocyte counts over time between patients who did or did not receive topical glucorticosteroids (GC) at the 144 µg/d (**A**) and 172 µg/d (**B**) dose levels in the SC arm of the AMG 212 clinical study. Monocyte counts were tabulated over time, before and during AMG 212 SC dosing. “C” refers to cycle and “D” refers to day. “PRE” refers to predose. Each symbol/connecting line represents individual subjects who did apply topical GC (+GC) or did not (-GC) on their injection sites. The apparent decrease in monocyte count from Day -7 to Cycle 1 Day 1, during which clobetasol propionate is administered, may be confounded by the prophylactic Dexamethasone to mitigate against Cytokine Release Syndrome (CRS) before the start of dosing in these subjects. To determine whether GC impacted peripheral blood monocyte counts over time, an unpaired t test was applied to the data comparing both groups at each time point. At the 144 ug/d cohort, monocyte counts of +GC (n=3) compared to -GC (n=3) subjects showed a *p-value* of 0.28, 0.15 and 0.44 at the screening, Cycle 1 Day 1 (C1D1) predose and Cycle 1 Day 15 (C1D15) predose timepoints respectively. At the 172 ug/d cohort, monocyte counts of +GC (n=6) compared to -GC (n=3) subjects showed a *p-value* of 0.43, 0.15 and 0.99 at the screening, C1D1 predose and C1D15 predose timepoints respectively. Collectively, the data show that the use of topical GC did not significantly change peripheral blood monocyte counts.
